# *Vibrio parahaemolyticus* and *Vibrio vulnificus in vitro* colonization on plastics influenced by temperature and strain variability

**DOI:** 10.3389/fmicb.2022.1099502

**Published:** 2023-01-10

**Authors:** Ryan E. Leighton, Karlen Enid Correa Vélez, Liyan Xiong, Addison G. Creech, Karishma P. Amirichetty, Gracie K. Anderson, Guoshuai Cai, R. Sean Norman, Alan W. Decho

**Affiliations:** ^1^Department of Environmental Health Sciences, University of South Carolina, Columbia, SC, United States; ^2^NIEHS Center for Oceans and Human Health and Climate Change Interactions, University of South Carolina, Columbia, SC, United States

**Keywords:** *Vibrio vulnificus*, *Vibrio parahaemolyticus*, biofilms, strain variability, extracellular polymeric substances, plastics, climate change

## Abstract

Marine bacteria often exist in biofilms as communities attached to surfaces, like plastic. Growing concerns exist regarding marine plastics acting as potential vectors of pathogenic *Vibrio*, especially in a changing climate. It has been generalized that *Vibrio vulnificus* and *Vibrio parahaemolyticus* often attach to plastic surfaces. Different strains of these *Vibrios* exist having different growth and biofilm-forming properties. This study evaluated how temperature and strain variability affect *V. parahaemolyticus* and *V. vulnificus* biofilm formation and characteristics on glass (GL), low-density polyethylene (LDPE), polypropylene (PP), and polystyrene (PS). All strains of both species attached to GL and all plastics at 25, 30, and 35°C. As a species, *V. vulnificus* produced more biofilm on PS (*p* ≤ 0.05) compared to GL, and biofilm biomass was enhanced at 25°C compared to 30° (*p* ≤ 0.01) and 35°C (*p* ≤ 0.01). However, all individual strains’ biofilm biomass and cell densities varied greatly at all temperatures tested. Comparisons of biofilm-forming strains for each species revealed a positive correlation (*r* = 0.58) between their dry biomass weight and OD_570_ values from crystal violet staining, and total dry biofilm biomass for both species was greater (*p* ≤ 0.01) on plastics compared to GL. It was also found that extracellular polymeric substance (EPS) chemical characteristics were similar on all plastics of both species, with extracellular proteins mainly contributing to the composition of EPS. All strains were hydrophobic at 25, 30, and 35°C, further illustrating both species’ affinity for potential attachment to plastics. Taken together, this study suggests that different strains of *V. parahaemolyticus* and *V. vulnificus* can rapidly form biofilms with high cell densities on different plastic types *in vitro*. However, the biofilm process is highly variable and is species-, strain-specific, and dependent on plastic type, especially under different temperatures.

## Introduction

*Vibrio parahaemolyticus* and *Vibrio vulnificus* are two known marine pathogens that naturally exist in the marine environment and can infect both marine animals and humans (Baker-Austin et al., 2018). They are a major concern to human health as they commonly infect humans through consumption of raw seafood (Elmahdi et al., 2018). The Centers for Disease Control and Prevention (CDC) estimates that pathogenic *Vibrio* cause approximately 80,000 illnesses in the United States each year, with 52,000 of these cases likely being attributed to ingestion of contaminated seafood (Centers for Disease Control and Prevention, 2019). However, the exact number of cases of vibriosis is unknown due to underreporting in clinical settings, as a typical infection can present as symptoms like other common health problems (Baker-Austin et al., 2010; Bell and Bott, 2021). Symptoms of both *V. parahaemolyticus* and *V. vulnificus* infections include cramps, nausea, fever, and bloody diarrhea. *V. vulnificus* skin infections can be more severe and lead to rapid septicemia and death if an open wound encounters salt or brackish water (Centers for Disease Control and Prevention, 2019). Most bacterial diseases in humans are caused by biofilm infections, which are bacteria embedded within a self-secreted matrix that offers protection from the outside environment (Jamal et al., 2018).

Marine bacteria, including potentially pathogenic *Vibrio* species, often exist in biofilms, where communities of microbes are enclosed in a protective, self-secreted matrix of extracellular polymeric substances (EPS) and attached to a surface or as suspended aggregates (Decho and Gutierrez, 2017). The EPS matrix consists of organic polymers such as polysaccharides, proteins, and eDNA (extracellular DNA), and protects bacteria from environmental stresses like desiccation, changes in temperature and pH, competition and predation, sunlight exposure, and from low nutrient conditions (De Kievit et al., 2001; Stewart and William Costerton, 2001; Donlan, 2002; Zettler et al., 2013; Decho and Gutierrez, 2017; Lami, 2019). This matrix also contributes to enhanced protection of pathogenic strains from antibiotics and enhances virulence (Schroeder et al., 2017). In the past, most studies of bacteria have focused on analyses of individual planktonic cells in the water column. However, many natural marine bacteria, like *Vibrio*, often exist in biofilm states. Biofilms commonly occur on a variety of substrates in marine environments including animal carapaces, algae, ship hulls, and specifically plastics (Zettler et al., 2013; De Tender et al., 2015; Dang and Lovell, 2016; Lage and Graca, 2016; de Carvalho, 2018). Growth of biofilms can be influenced by environmental factors including temperature.

Temperature is a primary environmental variable that influences *Vibrio* planktonic and biofilm lifecycles, and contributes greatly to growth and habitat range (Gilbert et al., 2012; Tiruvayipati and Bhassu, 2016; Ward et al., 2017; Hernández-Cabanyero et al., 2020). This presents a possibility that bacterial cells enclosed in the biofilm matrix on plastic surfaces may be responding to environmental changes by exhibiting different growth and activity patterns compared to their planktonic counterparts (Guzmán-Soto et al., 2021). Most cases of vibriosis occur during summer months due to warmer sea surface temperatures in which the bacteria thrive. However, *V. parahaemolyticus* and *V. vulnificus* infections are increasing in prevalence due to climate change contributing to rising seawater temperatures and extending the length of time of warm sea surface temperatures (Parry et al., 2007; Whitehead et al., 2009; Baker-Austin et al., 2013, 2016; Vezzulli et al., 2016; Deeb et al., 2018; Centers for Disease Control and Prevention, 2019). Since these two *Vibrio* species are known to form biofilms and have been shown to be early colonizers of plastic surfaces, it follows that plastics could increase *Vibrio* exposure to humans (Kesy et al., 2021; Tavelli et al., 2022). Attached biofilms could contribute to higher bacterial concentrations in contaminated seafood, leading to increased levels of bacterial exposure to humans if consumed raw (Keswani et al., 2016; Kesy et al., 2021).

The hydrophobic or hydrophilic nature of the bacterial cell surface also plays a major role in bacteria’s ability to colonize and form biofilms on abiotic surfaces like plastics (Rosenberg, 1984; Reifsteck et al., 1987). More hydrophobic cells adhere more strongly to hydrophobic surfaces like plastic, while more hydrophilic cells adhere more strongly to hydrophilic surfaces like glass (Kochkodan et al., 2008; Giaouris et al., 2009). It is generally accepted that the lifecycles of pathogenic *Vibrios*, like *V. parahaemolyticus* and *V. vulnificus,* include natural environmental and host-associated stages (Kamp et al., 2013; Tiruvayipati and Bhassu, 2016; Ghenem et al., 2017; Hernández-Cabanyero and Amaro, 2020). It has been suggested that within marine environments, exposure to changes in temperature may increase the chances of survival and infectivity of *V. parahaemolyticus* and *V. vulnificus* within host-associated stages (Motes et al., 1998; Strom and Paranjpye, 2000; Froelich and Noble, 2016; Sullivan and Neigel, 2018). While studies have identified genotypic and phenotypic traits that allow these bacteria to survive within each environment, the ability to form biofilms on plastics, which could help the bacteria transition between the two environments by ingestion, is not well understood (Reidl and Klose, 2002; Oberbeckmann et al., 2015; Hernández-Cabanyero et al., 2019). There is an underlying knowledge gap regarding hydrophobicity of different strains of *V. parahaemolyticus* and *V. vulnificus* and their interactions with different types of plastics.

Bacterial colonization and biofilm development on surfaces involve multiple processes, one of which is material-type surface characteristics (Flemming, 2016). This means that hydrophobicity, hydrophilicity, and chemical composition of a surface like plastic can influence bacterial attachment and development (Nakanishi et al., 2021). There are several major types of plastic, which include polyethylene (PE), polypropylene (PP), and polystyrene (PS). There are increased probabilities that these plastic types end up in marine environments due to their high production and usage (Andrady, 2003, 2011; Brien, 2007; Eriksen et al., 2014; Lusher et al., 2017). Contamination of marine habitats by large pieces of plastics (macroplastics) has raised environmental concerns due to their possible transfer to animals that may coincidently or selectively ingest plastic particles that have been mistaken for food, leading to health complications and death (Gregory, 2009). In addition, plastics poorly degrade in marine environments, and this degradation leads to smaller particulates, deemed “microplastics,” which are classified as plastic particles smaller than 5 mm in size (Arthur et al., 2009; Eriksen et al., 2014; GESAMP, 1997). There are growing concerns that both macro- and micro-plastics can travel large distances and act as transport vectors for attached bacterial pathogens (Zettler et al., 2013; Oberbeckmann et al., 2015; Debroas et al., 2017; Kesy et al., 2019; Bowley et al., 2021).

Microbial communities associate and live on plastic surfaces in the marine environment. These plastic-associated communities have been termed the “Plastisphere,” and have raised serious implications for both marine life and human health (Ward and Kach, 2009; Zettler et al., 2013). *Vibrio* have been found to be a major community member on marine plastic particles, but *Vibrio* concentrations on plastic surfaces have appeared lower compared to natural marine particles (Bryant et al., 2016; Amaral-Zettler et al., 2020; Curren et al., 2020). However, since *Vibrio* biofilms have still been found on numerous macro- and micro-plastic substate surface types in several marine surface waters, this implies that plastic particles could act as transport vectors of potentially pathogenic *Vibrio* to new areas outside of their native range and to marine animals that may accidently or selectively ingest the biofilm-associated plastic particles coincidently with food particles (Goldstein et al., 2014; Reisser et al., 2014; Kirstein et al., 2016; Viršek et al., 2017; Bowley et al., 2021). In addition, since these bacteria are in close proximity to each other in biofilms on plastics, there is high potential for horizontal transfer of antibiotic-resistance genes, compounding the exposure risk to both marine and human health (Arias-Andres et al., 2018; Laverty et al., 2020).

In this study, we examined the effect of temperature on *in vitro* biofilm production by *V*. *parahaemolyticus* and *V. vulnificus* on different types of plastics, which included low-density polyethylene, polypropylene, and polystyrene. We compared biofilm production of both species, from three strains isolated from different sources (human, animal, and water), a total of six different strains. We hypothesized that all strains from both *Vibrio* species would produce greater amounts of biofilm on all plastic types compared to a glass (control) due to the increased hydrophobic properties of plastic, which make it a more suitable substrate for colonization. Higher temperatures for *V. parahaemolyticus* and lower temperatures for *V. vulnificus* should also lead to increased biofilm formation on plastics due to previous studies that have examined both species’ biofilm production under different temperature conditions. We also postulated that human isolated strains of both species would produce the greatest amount of biofilm on all plastic types compared to animal and seawater isolated strains due to the harsher survival conditions in human hosts compared to the marine environment.

## Materials and methods

### Bacterial strains and growth conditions

Two clinical and two animal strains were obtained from the American Type Culture Collection (ATCC, Manassas, VA, United States). One seawater strain was gifted from the National Oceanic Atmospheric Administration (NOAA), Charleston, SC, United States, and was originally isolated from the marine environment (methods in Supplementary material, Vickery et al., 2007) in South Carolina, and one other seawater strain for this study was also directly isolated from the marine environment (methods in Supplementary material, Kim et al., 2015) in South Carolina (Table 1). *Vibrio parahaemolyticus* strains are commonly classified by their species marker (*tlh*) and capacity to infect humans through production of thermostable direct hemolysin (*tdh*) or thermostable direct hemolysin-related hemolysin (*trh*) virulence factors (Honda and Iida, 1993; Broberg et al., 2011). In our study, human isolated strain ATCC17802 contained *tlh* and *trh,* mollusk isolated strain ATCC43996 contained *tlh* and *tdh,* while the seawater isolated strain vpC12 only contained the species marker *tlh*. While *V. vulnificus* strains can also be classified by virulence factors, *V. vulnificus* can also be classified by 16S rRNA typing, which reveals if they are more clinically (type B, higher possible human infectivity) or more environmentally (type A, higher possible marine vertebrate infectivity) associated. In our study, the human isolated strain ATCC27562 and seawater isolated strain are type B, while eel isolated strain (ATCC33147) is type A.

**Table 1:** *Vibrio* strains used in this study.

Species	Isolation source	Strain ID	Isolate origin	Characteristics
*V. parahaemolyticus*	ATCC	ATCC17802	Human	*tlh*/*trh*
*V. parahaemolyticus*	ATCC	ATCC43996	Mollusk	*tlh*/*tdh*
*V. parahaemolyticus*	UofSC	vpC12	Seawater	*tlh*
*V. vulnificus*	ATCC	ATCC27562	Human	16S Type B
*V. vulnificus*	ATCC	ATCC33147	Eel	16S Type A
*V. vulnificus*	NOAA	vv155	Seawater	16S Type B

One clinical, one animal, and one seawater isolated strains of both *V. vulnificus* and *V. parahaemolyticus* were tested for biofilm formation at different temperatures on different substrate surfaces. All strains were maintained in 25% (*v*/*v*) glycerol at −80°C to be used in further experiments. A single colony of each bacteria was inoculated in 5 ml modified seawater with yeast extract (MSYE, ATCC medium 804, Oliver and Colwell, 1973) broth supplemented with calcium chloride (1.8 g/l), as calcium chloride contributes to biofilm formation (Tischler et al., 2018), and incubated overnight at 35°C with shaking (180 rpm). After incubation, the broth culture was adjusted to 10^7^ cells (OD_600_) using a SpectraMax M3 plate reader after calibrating the instrument’s absorbance values to cell counts from spread plating (Molecular Devices, San Jose, CA, United States).

### Biofilm formation

Biofilm formation experiments were adapted from Hamanaka et al. (2012) and Valquier-Flynn et al. (2017). Disc coupons (Table 2, BioSurface Technologies, Boseman, MT, United States) were chemically sterilized (70% ethanol for GL and PP, 70% isopropanol for LDPE and PS) for 24 h and were then placed in sterile Petri dishes in a biosafety cabinet until residual alcohol evaporated. Then, the coupons were placed in 24-well sterile non-treated microplates (Costar®, Corning, NY, United States) or sterilized slide coupons (Biosurface Technologies) in sterile Petri dishes (Falcon^®^, Corning, NY, United States), and then were filled with 990 μl (24-well microplate) or 14.85 ml (Petri dish) of fresh MSYE broth supplemented with calcium chloride medium. The plates were then inoculated with 10 μl of the bacterial cultures for 24 well plates and 150 μl for Petri dishes (10^7^ cells) to achieve a final cell density of 10^5^ cells per well/dish. Then, the 24-well plates were incubated at 25, 30, and 35°C with low shaking (125 rpm) to form biofilms in 24 h, and Petri dishes were incubated at 30°C with low shaking (85 rpm) to form biofilms in 48 h, with spent media in Petri dishes being replaced with 15 ml fresh media after 24 h. Low shaking conditions, instead of static, were chosen to introduce shear stress to the biofilms, to better resemble the marine environment. Borosilicate glass coupons were chosen as the substrate type controls and used as the substrate reference for statistical analyses. Wells/dishes containing MSYE broth supplemented with calcium chloride without inoculation and with coupons were used as blank and group controls. Biofilm biomass on each disc coupon experimental and control group had biological triplicates and each experiment was conducted three times independently. Biofilm biomass on each slide coupon experimental group was pooled from 10 biological replicates one time. All plates/dishes were sealed with Parafilm^™^ (Bemis, Neenah, WI, United States) to reduce evaporative loss of media.

**Table 2:** Coupon types and characteristics used in this study.

Coupon type	Chemical formula	Density	Diameter or length/thickness	Surface area	Usage
Borosilicate glass	BH_6_NaO_7_Si	Disc coupon: 2.19 g/cm^3^Slide coupon: 2.48 g/cm^3^	Disc coupon: 12.7 mm/3.8 mmSlide coupon: 75 mm/1 mm	Disc coupon: 405 mm^2^Slide coupon: 2,460 mm^2^	Laboratory and kitchen glassware, industrial systems, electronics
Low-density polyethylene	(C₂H₄)_n_	Disc coupon: 0.89 g/cm^3^Slide coupon: 0.86 g/cm^3^	Disc coupon: 12.7 mm/3.8 mmSlide coupon: 73 mm/1.6 mm	Disc coupon: 405 mm^2^Slide coupon: 2,501 mm^2^	Plastic bags, six-pack rings, packaging film, bottles, netting
Polypropylene	(C_3_H_6_)_n_	Disc coupon: 0.87 g/cm^3^Slide coupon: 0.83 g/cm^3^	Disc coupon: 12.7/3.8 mmSlide coupon: 75/1.6 mm	Disc coupon: 405 mm^2^Slide coupon: 2,569 mm^2^	Rope, bottle caps, packaging film, netting
Polystyrene	(C_8_H_8_)_n_	Disc coupon: 1.05 g/cm^3^Slide coupon: 1.18 g/cm^3^	Disc coupon: 12.7/3.8 mm Slide coupon: 77 mm/0.6 mm	Disc coupon: 405 mm^2^Slide coupon: 2,451 mm^2^	Plastic utensils, food containers

### Crystal violet staining assay

Biofilms of both *V. parahaemolyticus* and *V. vulnificus* were quantified by crystal violet staining according to O’Toole, (2011) and Valquier-Flynn et al. (2017) with some modifications. Following 24-h incubation, planktonic cells were removed from the 24-well microplates before gently washing with 1 × phosphate buffer saline (PBS, Molecular Biologicals International, Irvine, CA, United States) three times. 500 μl of 100% methanol (Sigma-Aldrich) was then added to the plates to fix the biofilms to the glass and plastics and incubated at room temperature for 20 min. Then, the methanol was removed, and residual methanol was allowed to evaporate off disc coupon surfaces. The biofilms were stained with 700 μl of 0.1% (*w*/*v*) crystal violet (Sigma-Aldrich) for 15 min at room temperature. The staining solution was removed, and then 1 × PBS was used to remove the non-bound dye four times. The glass and plastic coupons were then transferred to a new 24-well non-treated microplate and the stained and washed biofilms were air-dried overnight. Lastly, 600 μl of 30% acetic acid (Fisher Scientific) was added to dissolve the bound crystal violet and incubated at room temperature for 15 min. Optical densities of each well were measured by absorbance (570 nm) using a SpectraMax M3 plate reader (Molecular Devices). Mean OD_570_ values were then divided by the surface area (405 mm^2^) of the disc coupons tested to obtain final biofilm biomass values per mm of the surface type.

### Biofilm removal and determination of colony counts

Total colony counts were determined from biofilm suspensions according to Portillo et al. (2013) and Bjerkan et al. (2009) with some modifications. Following 24-h incubation, planktonic cells were removed from the 24-well non-treated microplate wells before washing disc coupons with 700 μl 1 × PBS gently, four times. Then, disc coupons were placed individually in 10 ml 1 × PBS in a conical tube (Falcon^®^) and vortexed using a Vortex Genie 2^®^ (Fisher Scientific) at the highest setting for 1 min. Then, coupons and 1 × PBS solution were individually transferred to borosilicate glass culture tubes (VWR International, Radnor, PA, United States) and placed in a Branson M2800 ultrasonication water bath (Branson Ultrasonics, Brookfield, CT, United States) and sonicated for 5 min at 40 kHz. The coupons and 1 × PBS solution were then transferred back to conical tubes, and vortexed again for 1 min. Then, the biofilm suspension in 1 × PBS was serially diluted in 1X PBS in conical tubes and 10^−4^ to 10^−7^ serial dilutions were spread onto prewarmed MSYE supplemented with calcium chloride agar plates. Plates were incubated at 30°C for 20–24 h. The viability of cells was determined in terms of colony-forming units (CFU) per coupon. Biofilm cell densities of each disc coupon experimental and control group had biological triplicates and each experiment was conducted three times independently. Mean CFU values were then log transformed and divided by the surface area (405 mm^2^) of the disc coupons tested to obtain final CFU values per mm of the surface type.

### Extracellular polymeric substance extraction and measurements of dry cell and EPS biomass concentrations

The strains of both *Vibrio* species that exhibited the greatest biofilm biomass, on average, combined on all plastic disc surface types were used for measuring cell and EPS concentrations. *V. parahaemolyticus* strain ATCC17802 (human) and *V. vulnificus* strain vv155 (seawater) exhibited the greatest mean combined biomass per mm^2^ of all plastic disc surfaces at 30°C (OD_570_/405mm^2^ ~ 4.17*E*-03). EPS extraction was conducted according to Bramhachari et al. (2007) with some modifications. Following 48-h incubation at 30°C, planktonic cells were removed from Petri dishes before washing slide coupon with 10 ml 0.85% saline gently two times. Then, the slide coupon was placed in 30 ml 0.85% saline in a conical tube and vortexed at highest setting for 1 min for plastics, and low setting for glass. Then, coupon and 0.85% saline solution were transferred to borosilicate glass test tube, and placed in water sonication bath, and sonicated for 5 min at 40 kHz. The coupon and saline solution were then transferred back to conical tube, and vortexed again for one minute. Lastly, the coupon was then scraped on all sides with a cell scraper (Falcon^®^, Corning, NY, United States), scraper submerged in solution, and coupon was removed. This was repeated 9 more times to pool 10 slide coupons’ total cell and EPS contents in 0.85% saline solution. Then, the 30 ml 0.85% saline biofilm suspension was centrifuged (4000 × *g*) to pellet cells. Cell pellet was then resuspended in the same solution, centrifuged again, and this process was repeated two more times. Cell pellet was saved at 4°C, while supernatant (EPS solution) was then immediately mixed with 75% total volume cold ethanol (VWR) overnight to precipitate the EPS. Total EPS and ethanol solution were then centrifuged to pellet EPS, the supernatant was removed, and the remaining EPS saved. The cell pellet and crude EPS were then freeze-dried using a FreeZone^®^ 6 system (Labconco, Kansas City, MO, United States) and weighed.

### Extracellular polymeric substance chemical composition analysis

The total carbohydrate content was measured after first dialyzing the EPS solution in SnakeSkin^™^ membrane with a 10,000 molecular weight cut-off (Fisher Scientific) in a borosilicate glass beaker of deionized water for 24 h at 4°C. Then the EPS solution was mixed with 75% total volume cold ethanol (VWR) overnight to precipitate the EPS. Total EPS and ethanol solution were then centrifuged to pellet EPS, the supernatant was removed, and the remaining EPS was saved. The EPS was then freeze-dried and weighed. This was repeated three times for each plastic type for (1) carbohydrate, (2) protein, and (3) eDNA quantification. (1) Dried crude EPS was prepared and carbohydrate content was quantified according to Dubois et al. (1951) using a Total Carbohydrate Assay Kit with glucose as the calibration standard according to the manufacturer’s instructions (Cell Biolabs, San Diego, CA, United States). The measurement was carried out using absorbance (490 nm; Molecular Devices, San Jose, CA, United States). (2) Dried crude EPS was prepared by using a Compat-Able^™^ Protein Assay Preparation Reagent kit (Fisher Scientific) according to Jiao et al. (2010) and manufacturer’s instructions. Then, the protein content was measured using a Bradford assay kit with bovine serum albumin (BSA) as the calibration standard according to the manufacturer’s instructions (Fisher Scientific). Absorbance measurements were conducted (595 nm). (3) Dried crude EPS was prepared according to Grande et al. (2015). EPS was resuspended in 1 ml 1X TE buffer (Fisher Scientific) and DNA was quantified using the Invitrogen Quant-iT^™^ PicoGreen^™^ dsDNA reagent kit (Molecular Probes, Eugene, OR, United States), with λ-DNA as the calibration standard according to the manufacturer’s instructions. Fluorescence was measured using a SpectraMax M3 plate reader (excit/emiss = 480/520 nm). % EPS by weight was calculated by standardization of each mean concentration of proteins, carbohydrates, and eDNA to μg/ml, then divided by total starting weight of pooled crude EPS from ten samples.

### Hydrophobicity assay

Microbial adherence to hydrocarbons was determined using p-xylene according to the MATH test method (Rosenberg, 1984; Kwaszewska et al., 2006; Mizan et al., 2016) with slight modifications. Briefly, overnight cultures of all strains in MSYE broth supplemented with calcium chloride were diluted to 10^5^ cells and then grown at 25, 30, and 35°C for 24 h at 125 rpm. The cells were harvested by centrifugation (4000 × *g*) for 10 min, washed twice with 1 × PBS, and then resuspended in 1 × PBS to an OD_600_ ~ 0.3–0.6 (A0). One milliliter of p-xylene (Beantown Chemical, Hudson, NH, United States) was added to a conical tube containing four mL of the adjusted bacterial/PBS suspension and the mixture was then vortexed vigorously at the highest setting for two minutes and incubated for 20 min at room temperature to allow separation of the two phases. The supernatant (aqueous hydrocarbon phase) was then carefully removed using glass Pasteur pipettes and cellular absorbance was measured (OD_600_) in PBS suspension (A1). Hydrophobicity was calculated as the percentage of planktonic cells partitioning into the hydrocarbon phase. The percentage of p-xylene partitioning was estimated using the following formula: ([A0–A1]/A0) × 100 (Rivas et al., 2008). A mean adherence to p-xylene ≤ 30% indicated that the strains were hydrophilic; values > 30% signified hydrophobic strains. Highly hydrophobic strains exhibited values ≥ 70% (Kwaszewska et al., 2006). Each experimental and control group was completed in biological triplicate and each experiment was conducted independently three times.

### Statistical analyses

The experimental data for biomass CFUs and hydrophobicity were expressed as the mean ± standard deviation. Biomass dry weights from slide coupons were expressed as mean total pooled biomass from 10 biological replicates. Biochemical characteristic weights of EPS were expressed as a percentage of the total pooled EPS weight of plastic type. Two-way analysis of variance (ANOVA) models were calculated using Rstudio software to compare value differences (*α* = 0.05). Strain, temperature, and surface type were the variables for all models. Glass was selected as the reference surface and 25°C was selected as the reference temperature for all analyses. Also, *V. parahaemolyticus* strain ATCC17802 was selected as the reference strain for all *V. parahaemolyticus* strains while *V. vulnificus* strain ATCC27562 was selected as the reference strain for all *V. vulnificus* strains. Bonferroni correction was calculated and applied to *p*-values to control for type 1 error. A *t*-test (*α* = 0.05) was calculated for comparison between mean total dry biomass weights between all plastics and glass and a Pearson’s correlation coefficient was calculated for comparison between mean total dry biomass weight and mean biofilm biomass absorbance data using Excel’s data analysis toolpak.

## Results

### Plastics enhance *Vibrio parahaemolyticus* and *Vibrio vulnificus* biofilm formation compared to glass

Experiments were conducted to test the effect of temperature (25, 30, and 35°C) on biofilm biomass production and biofilm cell viability on glass (GL), low-density polyethylene (LDPE), polypropylene (PP), and polystyrene (PS) by three different strains of both *V. parahaemolyticus* and *V*. *vulnificus* for 24 h. The vortex/sonication method as described previously was first tested to confirm efficacy of biofilm removal from all substate surfaces while also preserving cell viability (Supplementary Figures S1–S3; Supplementary Tables S14, S15). Raw mean data are presented in Supplementary Tables S1, S6.

**Figure 1:**
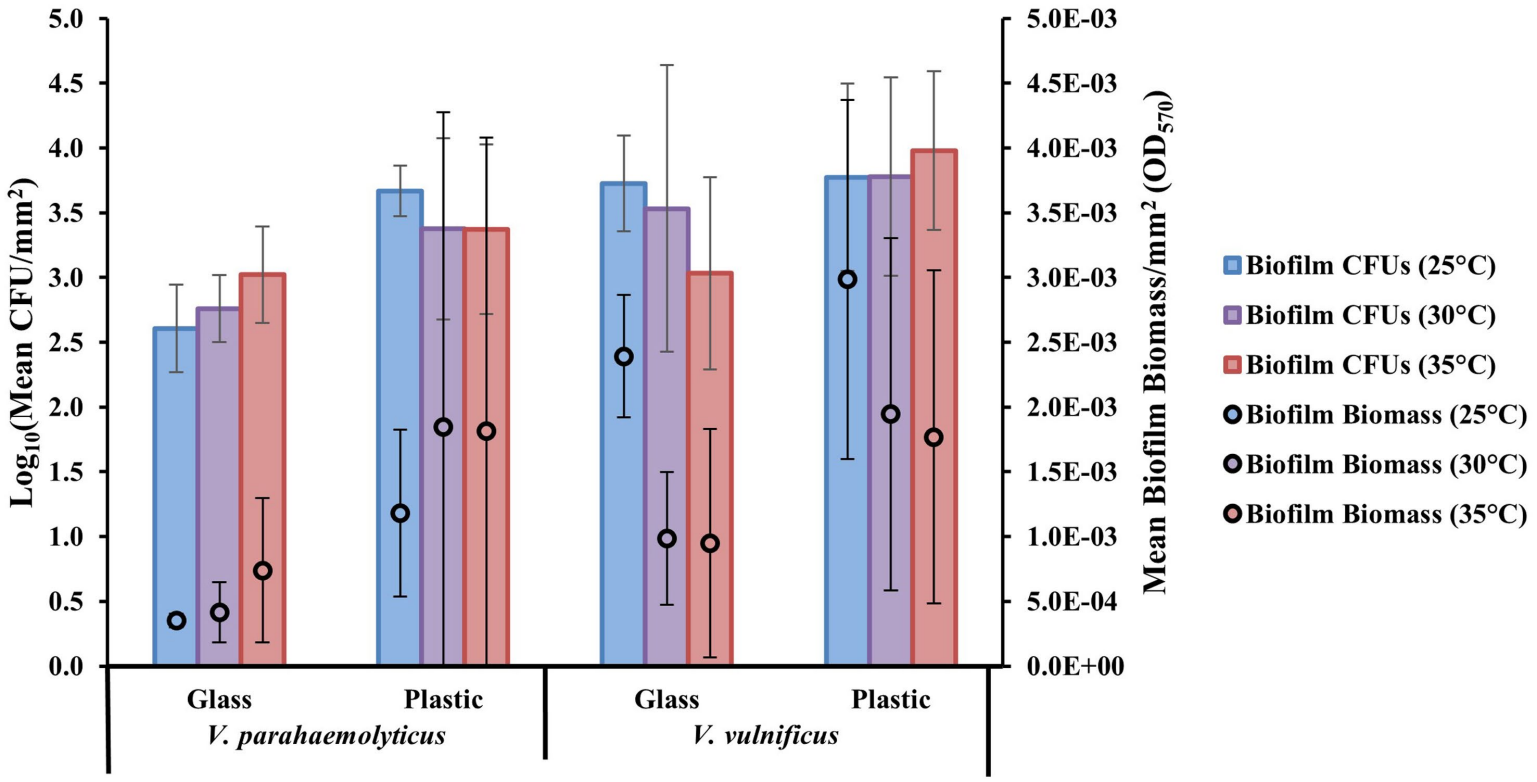
Plastics enhance *Vibrio parahaemolyticus* and *Vibrio vulnificus* biofilm formation compared to glass. Effect of temperature (°C) on mean biofilm biomass and colony-forming units (CFUs; means ± SD) by *V. parahaemolyticus* and *V. vulnificus* between glass and all plastics after 24 h (means of all biological triplicates and three independent experiments).

The crystal violet staining assay reflects total bacterial biomass formed on the substate surface types. The biofilm removal and colony count assay reflects biofilm cell densities (expressed as colony-forming units, CFUs) on the substate surface types. From these two assays, it was shown that at a species level, both *Vibrio parahaemolyticus* and *V. vulnificus* appeared to have greater biofilm biomass and CFU concentrations at all temperatures tested on all combined plastic types compared to GL (Figure 1). *V. parahaemolyticus* formed greater biofilms and had slightly greater biofilm CFU concentrations at 30 and 35°C on all combined plastic types. *V. vulnificus* formed greater biofilm biomass at 25°C, but had slightly greater biofilm CFU concentrations at 30 and 35°C. The comparison of biofilm biomass between *Vibrio* species revealed high biomass variability between substrate surface composition types (glass vs. plastic) at different temperatures, as indicated by high standard deviation bars. Comparison of biofilm biomass between combined species isolated types (human, animal, and water) also revealed high biomass variability between all substrate surfaces at different temperatures (Supplementary Figure S4). However, these high standard deviation bars are due to high variability between species and strain types.

### Surface material and temperature influences *Vibrio parahaemolyticus* and *Vibrio vulnificus* biofilm formation

**Figure 2:**
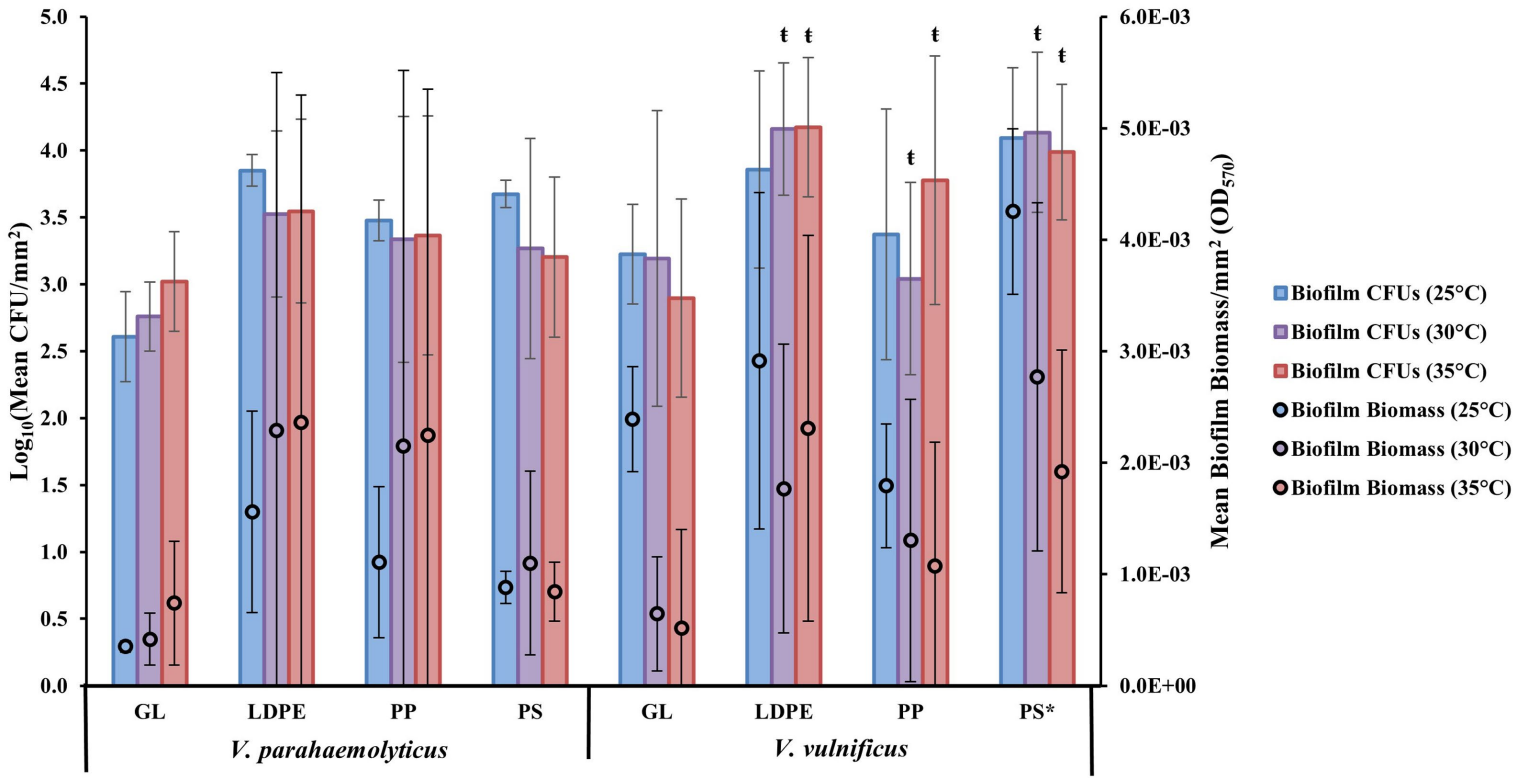
Surface material and temperature influence *V. parahaemolyticus* and *V. vulnificus* biofilm formation. Comparison of biofilm biomass and CFUs (means ± SD) by both *V. parahaemolyticus* and *V. vulnificus* between substate surface type at different temperatures after 24 h (means of all experiments). * = Significantly greater biofilm biomass compared to GL, ŧ = significantly less overall biofilm biomass compared to 25°C.

Examining biofilm formation on individual plastic types of LDPE, PP, and PS at different temperatures (25, 30, and 35°C) revealed that as a species *V. parahaemolyticus* appeared to form the greatest biofilms, on average, on LDPE and PP at 30 and 35°C and had higher CFU concentrations across all plastics compared to GL (Figure 2). *Vibrio parahaemolyticus* biofilm formation on PS was only marginally higher than GL at all temperatures yet still had an overall higher CFU concentration compared to GL. *Vibrio parahaemolyticus* formed the greatest biofilms across all temperatures on LDPE and PP, which have a specific density lower than seawater (~ 1.02), compared to PS and GL which have a higher specific density than seawater. Comparatively, as a species, *V. vulnificus* appeared to form greater biofilms, on average, on all plastic types at 25°C. Also, compared to *V. parahaemolyticus*, *V. vulnificus* biofilm formation was greatest on PS at 25°C. *Vibrio vulnificus* formed greater biofilms on LDPE at 35°C compared to 30°C, but this trend was opposite for PS as biofilm formation was greater at 30°C than at 35°C. *Vibrio vulnificus* biofilm biomass and CFU concentrations on LDPE and PS were also higher than GL across all temperatures. *Vibrio vulnificus* biofilm formation on PP was only slightly higher than GL at higher temperatures (30 and 35°C), and had lower biofilm biomass at 25°C and lower CFU concentrations on PP at 25 and 30°C compared to GL.

ANOVA revealed certain significant differences (*α* = 0.05) in the amount of biofilm formation on each plastic type compared to glass at the species level (Figure 2; Supplementary Tables S3–S5, S8–S10). *Vibrio parahaemolyticus* did not produce significantly more biofilm or significantly more CFUs on any plastic surface (*p* = 0.99) compared to GL. Temperature was also not a significant factor in contributing to *V. parahaemolyticus* biofilm biomass (30° *p =* 0.99, 35°C *p* = 0.99) or CFUs (30°C *p* = 0.99, 35°C *p* = 0.99). However, *V. vulnificus* produced significantly more biofilm, but not CFUs, on PS (*p* ≤ 0.05) compared to GL. *Vibrio vulnificus* biofilm biomass production was also significantly enhanced at 25°C compared to 30° (*p* ≤ 0.01) and 35°C (*p* ≤ 0.01).

### Strain type influences *Vibrio parahaemolyticus* and *Vibrio vulnificus* colonization and biofilm biomass and cell viability

**Figure 3:**
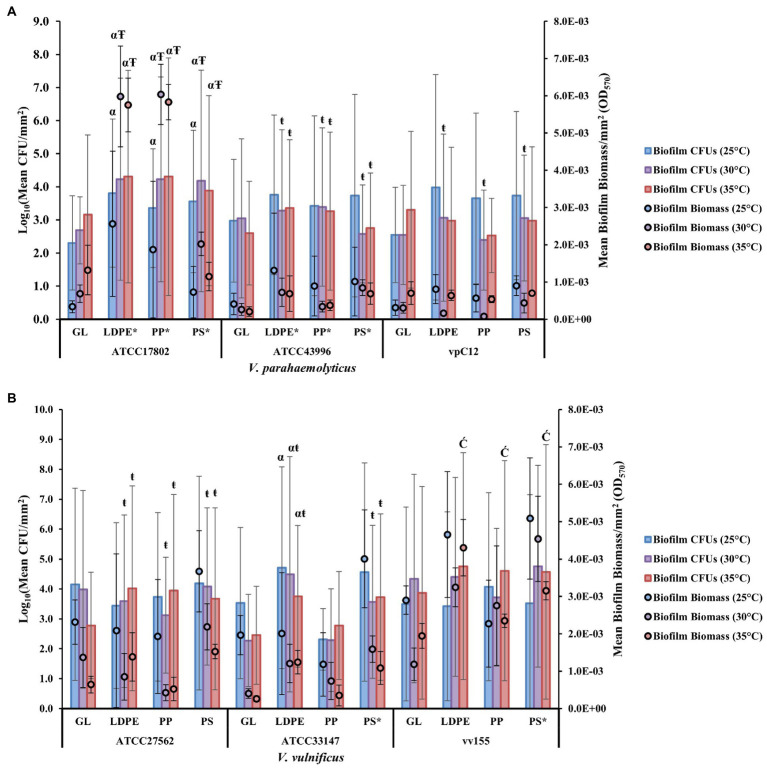
Strain type influences *V. parahaemolyticus* and *V. vulnificus* colonization and biofilm biomass and cell viability. Comparison of biofilm biomass and CFUs (means ± SD) by *V. parahaemolyticus* strains **(A)** and *V. vulnificus* strains **(B)** between glass and all plastic types at different temperatures after 24 h (means of all biological triplicates and three independent experiments).* = significantly greater biofilm biomass compared to GL, α = significantly greater CFUs compared to GL, Ŧ = significantly greater overall biofilm biomass compared to 25°C, ŧ = significantly less overall biofilm biomass compared to 25°C, Ć = significantly greater overall CFUs compared to 25°C.

At a strain level, the highest biofilm formation with a mean OD_570_ per mm^2^ (OD_570_/405mm^2^) of 5.92*E*-03 was obtained on LDPE and PP by *V. parahaemolyticus* strain ATCC17802 at 30°C, and the lowest biofilm formation with a mean OD_570_ per mm^2^ of 7.41E-05 was obtained on PP by *V. parahaemolyticus* strain vpC12 at 30°C. This further highlights the variability of biofilm formation between different strains of the same species (Figure 3A; Supplementary Table S1). All strains of both *Vibrio* species also had high concentrations of biofilm CFUs on GL and the three types of plastic over 24 h and under all temperature conditions. The highest CFU concentration was obtained on LDPE by *V. vulnificus* strain vv155 at 35°C, while the lowest CFU concentration was obtained on GL by *V. vulnificus* strain ATCC33147 at 30°C (Figure 3B; Supplementary Table S6). Further comparison of the individual strains revealed significant differences (*p* ≤ 0.05) in biofilm formation and CFUs between strains on different surfaces and temperatures (Figures 3A,B; Supplementary Tables S1, S6).

*Vibrio parahaemolyticus* animal isolate (ATCC43996) and seawater isolate vpC12 produced significantly lower biofilm biomass (*p* ≤ 0.001) and CFUs (*p* ≤ 0.01) than human isolate ATCC17802 (Supplementary Tables S2, S7). Human isolated strain ATCC17802 had significantly greater biofilm formation (*p* ≤ 0.05) and CFU concentrations (*p* ≤ 0.01) on all plastic surfaces compared to glass. This strain also produced significantly greater biofilms and had greater CFU concentrations at 30 and 35°C (*p* ≤ 0.05) compared to 25°C. Animal isolated strain ATCC43996 also had significantly greater (*p* ≤ 0.01) biofilm formation on all plastic surfaces compared to GL. However, elevated temperatures (30 and 35°C) significantly decreased (*p* ≤ 0.01) the amount of overall biofilm produced by this strain compared to 25°C, but an increase in temperature had no significant effect (30°C, *p* = 0.2; 35°, *p* = 0.12) on CFU concentrations. Seawater isolated strain vpC12 did not have significantly greater (*p* > 0.05) biofilm biomass or CFU concentrations on any plastic surface compared to GL; however, elevated temperature (30°C) did lead to a significant decrease (*p* ≤ 0.05) in overall biofilm production compared to 25°C.

*Vibrio vulnificus* animal isolate ATCC33147 had no significant differences in biofilm biomass (*p* = 0.99) or CFU concentrations (*p* = 0.99) compared to human isolate ATCC27562 (Supplementary Table S2, S7). However, water isolate vv155 surprisingly produced significantly greater biofilm biomass (*p* ≤ 0.001) and had significantly higher CFU concentrations (*p* ≤ 0.05) than ATCC27562. Human isolated strain ATCC27562 did not have significantly greater (*p* > 0.05) biofilm biomass or CFU concentrations on any plastic surface compared to GL; however, elevated temperature (30 and 35°C) did lead to a significant decrease (*p* ≤ 0.01) in overall biofilm production. Animal isolated strain ATCC33147 had significantly greater (*p* ≤ 0.05) biofilm formation on PS compared to GL, and significantly greater (*p* ≤ 0.05) CFUs on LDPE compared to GL. However, elevated temperature (30 and 35°C) also led to a significant decrease (*p* ≤ 0.05) in overall biofilm production, but not in CFU concentrations, compared to 25°C. Seawater isolated strain vv155 had significantly greater (*p* ≤ 0.05) biofilm formation on PS compared to GL, but an increase in temperature had no significant effect *(p* > 0.05) on overall biofilm biomass and CFU concentrations.

Comparison of biofilm biomass between combined species isolated sources (human, animal, and water) revealed high biomass and CFU variability between surface types at different temperatures as indicated by high standard deviations (Supplementary Figure S3). While it appeared human isolated strains tended to produce, on average, greater biofilms and CFUs on LDPE and PP at higher temperatures (30 and 35°C), *V. parahaemolyticus* strain ATCC17802 mainly accounted for this high biofilm mean due to it being the greatest biofilm former at higher temperatures compared to *V. vulnificus* strain ATCC27562 that formed greater biofilms at 25°C across all surface types (Figures 3A,B; Supplementary Tables S1, S6). The other strain sources (animal and water) of both species formed greater biofilms, on average, at 25°C across all surface types than at higher temperatures (30 and 35°C).

Comparison of biofilm biomass between isolates and plastic surface types revealed differences in mean percent change compared to GL (Table 3). 41/54 total means of biofilm biomass on plastic across all temperatures had a mean positive percent change in biofilm biomass compared to GL. The greatest mean positive percent change compared to GL was observed with strain ATCC17802 on LDPE and PP at 25 and 30°C. Strains vpC12 and ATCC27562 accounted for 9/12 of the negative mean percent changes in biomass across all temperatures, meaning that they formed greater biofilms on GL, on average, compared to plastic in these cases. However, most of these negative percent changes were attributed to LDPE and PP compared to GL, as both strains had a mean positive percent change on PS compared to GL.

**Table 3:** Summary of biofilm biomass showing percent change (%) between all *Vibrio parahaemolyticus* and *Vibrio vulnificus* strains and plastic types at different temperatures compared to glass controls.

Strain	Plastic type	25°C	30°C	35°C
ATCC17802	LDPE^*^	663%	774%	337%
	PP^*^	457%	782%	343%
	PS^*^	117%	195%	−13%
ATCC43996	LDPE^*^	220%	177%	236%
	PP^*^	118%	30%	83%
	PS^*^	148%	224%	234%
vpC12	LDPE	161%	−48%	−8%
	PP	83%	−74%	−23%
	PS	192%	42%	0%
ATCC27562	LDPE	−10%	−38%	116%
	PP	−17%	−69%	−18%
	PS	59%	60%	138%
ATCC33147	LDPE	2%	199%	378%
	PP	−40%	83%	35%
	PS*	104%	295%	318%
vv155	LDPE	61%	175%	121%
	PP	−22%	133%	21%
	PS^*^	75%	284%	62%

Green = (+), red = (−), yellow = no change. ^*^ = significantly greater overall biofilm formation on this surface compared to glass.

Comparison of biofilm CFU concentrations between isolates and plastic surface types at all temperatures tested revealed differences in % change compared to GL (Table 4). 42/54 total means of biofilm CFU concentrations on plastic across all temperatures had a mean positive % change in biofilm CFU concentrations compared to GL. The greatest mean positive % change compared to GL was observed with strain ATCC33137 at 30°C on LDPE. Strains vpC12 and ATCC27562 accounted for 8/12 of the negative mean % changes in biofilm CFUs across all temperatures, meaning that they had greater mean biofilm CFUs on GL compared to specific plastic types in these cases. However, strain vpC12 had a mean positive % change in CFU concentrations at 25°C on all plastic types, and strain ATCC27562 had a mean positive % change in CFU concentrations at 35°C on all plastic types. At 35°C, 5/6 strains had a mean positive % change in biofilm CFUs on all plastic types compared to glass and lower temperatures.

**Table 4:** Summary of biofilm CFUs showing percent change (%) between all *V. parahaemolyticus* and *V. vulnificus* strains and plastic types at different temperatures compared to glass controls.

Strain	Plastic type	25°C	30°C	35°C
ATCC17802	LDPE^*^	97%	97%	93%
	PP^*^	91%	97%	93%
	PS^*^	94%	97%	81%
ATCC43996	LDPE	84%	42%	83%
	PP	65%	55%	78%
	PS	83%	−196%	30%
vpC12	LDPE	96%	70%	−116%
	PP	92%	−43%	−499%
	PS	94%	69%	−116%
ATCC27562	LDPE	−418%	−147%	94%
	PP	−164%	−622%	93%
	PS	8%	20%	87%
ATCC33147	LDPE^*^	93%	99%	95%
	PP	−1,551%	3%	52%
	PS	91%	95%	95%
vv155	LDPE	−19%	13%	87%
	PP	74%	−324%	82%
	PS	6%	62%	80%

Green = (+), red = (−).

^*^significantly greater overall CFUs on this surface compared to glass.

### Differences in substrate type affect biofilm cell and extracellular polymeric substance concentration and composition

Across all strains, *V. parahaemolyticus* strain ATCC17802 and *V. vulnificus* strain vv155 had the highest mean combined biomass per mm^2^ of all plastics at 30°C (OD_570_/405mm^2^ ~ 4.17*E*–03). These strains were chosen to be further analyzed for cell and EPS weight and EPS biochemical characterization. Comparison of ATCC17802 and vv155 strains combined total dry biomass on glass compared to plastic revealed significantly greater total dry biomass weights on all plastic types compared to glass (*p* ≤ 0.01; Table 5). Further comparison revealed a moderately positive Pearson’s correlation coefficient (*r =* 0.58) between mean total dry biofilm biomass weights (mg) and mean biofilm biomass from crystal violet staining (OD_570_) of all surfaces of both strains.

**Table 5:** Estimated pooled cell and crude extracellular polymeric substance mean dry weight per slide coupon at 30°C after 48 h.

Strain	Coupon type	Mean dry cell weight (mg)	Mean dry crude EPS weight (mg)	Mean total dry biomass weight (mg)	Mean biofilm biomass (OD_570_)
ATCC17802	GL	0.03	0.04	0.07	0.27
	LDPE	0.09	0.09	0.18	2.42
	PP	0.07	0.1	0.17	2.44
	PS	0.04	0.05	0.09	0.81
vv155	GL	0.01	0.03	0.04	0.47
	LDPE	0.21	0.07	0.28	1.31
	PP	0.12	0.07	0.19	1.11
	PS	0.15	0.18	0.33	1.83
Significance (*p*), Correlation (*r*)				*p* ≤ 0.01	*r* = 0.58

Significance (*p*) was calculated by comparison of the mean total dry biomass weight between glass and all plastics combined. Correlation (*r*) was calculated by comparison of mean total dry biomass weights to respective mean biofilm biomass OD_570_ values.

**Figure 4:**
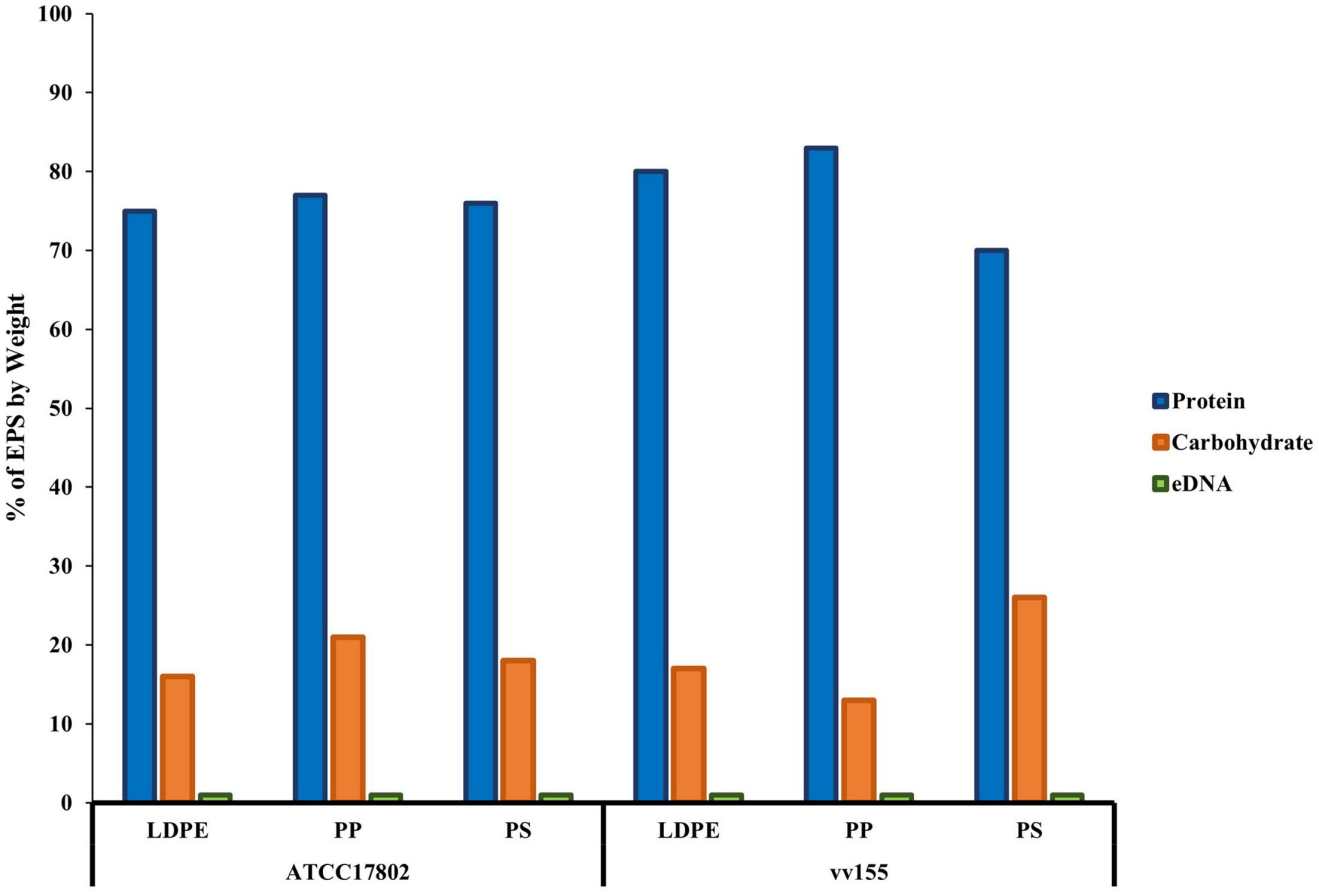
Proteins are the main component of *V. parahaemolyticus* and *V. vulnificus* extracellular polymeric substances on plastics. % extracellular polymeric substance (EPS) by weight of biochemical characteristics of *V. parahaemolyticus* (ATCC17802) and *V. vulnificus* (vv155) on all plastic types. % EPS by weight was calculated by standardization of each mean concentration of proteins, carbohydrates, and eDNA to μg/ml, then divided by total starting weight of pooled crude EPS from ten samples.

Biochemical characterization of both *Vibrio* species EPS revealed that extracellular proteins were the main component of the EPS, followed by carbohydrates and eDNA on all plastic types (Figure 4; Supplementary Table S11). *Vibrio parahaemolyticus* extracellular proteins accounted for 75, 77, and 76% of total EPS mass on LDPE, PP, and PS, respectively. *V. parahaemolyticus* extracellular carbohydrates made up 16, 21, and 18% of total EPS mass on LDPE, PP, and PS, respectively, and eDNA made up ~1% of total EPS mass on each plastic type. *V. vulnificus* extracellular proteins accounted for 80, 83, and 70% of total EPS mass on LDPE, PP, and PS, respectively. *Vibrio vulnificus* extracellular carbohydrates accounted for 17, 13, and 26% of total EPS on LDPE, PP, and PS, respectively, and eDNA also made up ~ 1% of total EPS mass on each plastic type.

### Temperature and strain variability influences planktonic cell hydrophobicity

**Figure 5:**
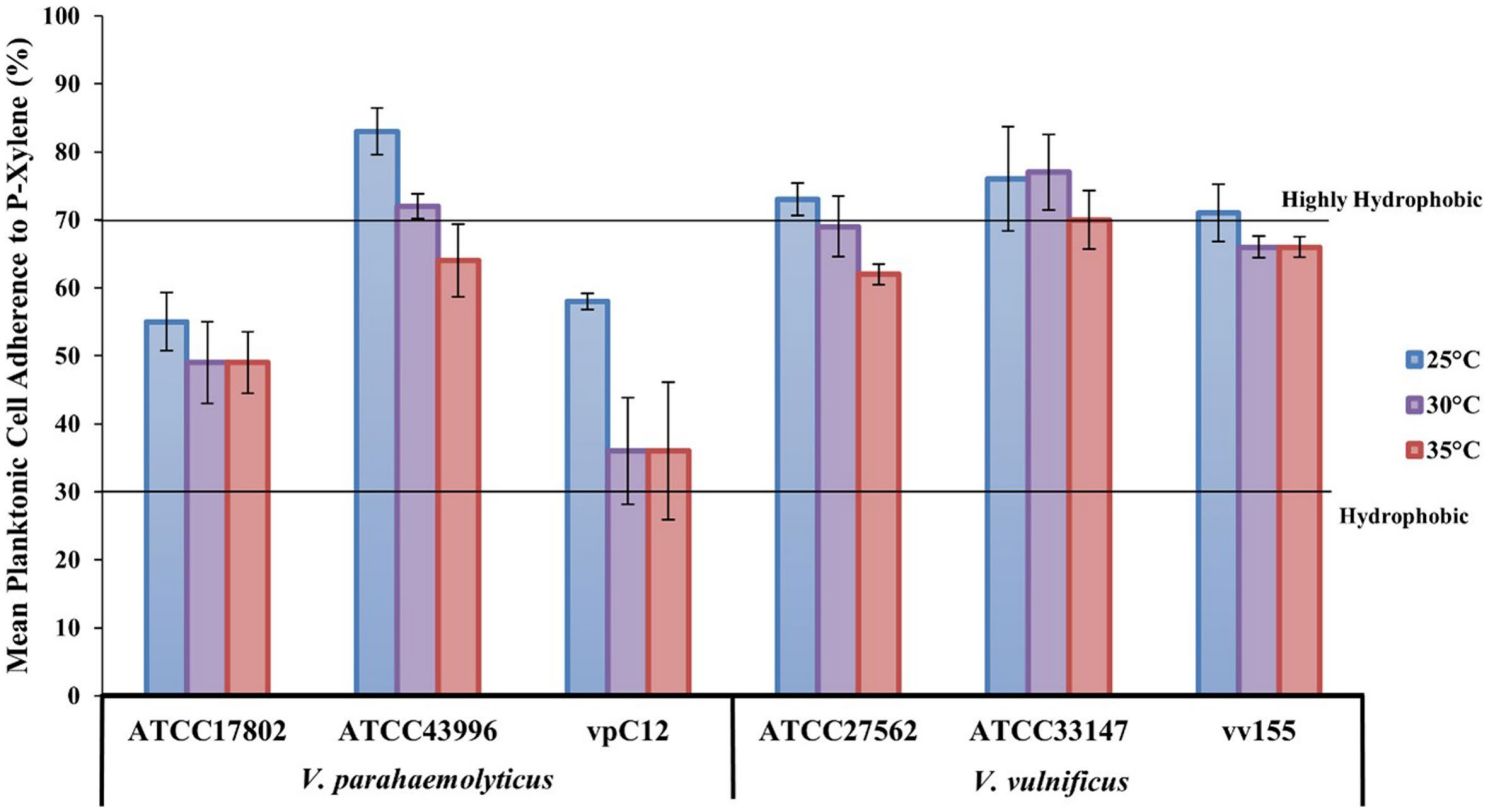
Lower temperatures increase *Vibrio* hydrophobicity. *V. parahaemolyticus* and *V. vulnificus* individual strain adherence (means ± SD) to p-xylene (%) at different temperatures (means of all biological triplicates and three independent experiments). Lines designate planktonic cell hydrophobicity from hydrophobic (30% adherence) to highly hydrophobic (70% adherence).

The MATH method, which is based on the degree of adherence to the hydrocarbon-p-xylene interface, showed that all strains were moderately (values > 30%) to strongly (values ≥ 70%) adhesive to p-xylene, and thus considered hydrophobic, at all temperatures tested (Figure 5). Raw mean hydrophobicity data are presented in Supplementary Table S12 in the Supplementary Data. Most strains (5/6) became slightly less hydrophobic as temperature increased from 25 to 35°C. *V. parahaemolyticus* strain ATCC43996 was highly hydrophobic at 25 and 30°C while all *V. vulnificus* strains were highly hydrophobic at 25°C, with strain ATCC33147 also being highly hydrophobic at 30 and 35°C. At a species level, *V. vulnificus* was, on average, more hydrophobic than *V. parahaemolyticus* at all temperatures tested, especially at 30 and 35°C (19 and 16% more hydrophobic, respectively; Supplementary Table S13).

## Discussion

While plastic pollution in the marine environment remains a global concern, their role as substrates for microbial habitats and subsequently vectors for the dispersion of pathogenic or non-pathogenic bacteria must be further evaluated, especially under evolving climate change scenarios (Zettler et al., 2013; Kirstein et al., 2016). *Vibrio parahaemolyticus* and *V. vulnificus* are potential pathogenic bacteria that can infect both marine animals and humans. In past decades, *Vibrio* habitat range has increased and coincided with an increase in plastic production and growth. This expansion of *Vibrio* coupled with their potential to colonize and live on numerous plastic types will increase the potential risk of both marine animal and human exposure to *Vibrio* species. To better understand the emerging environmental and public health risks associated with bacterial colonization of plastic particles, studies are needed to determine how this process is affected by different substrate types under different environmental conditions, such as temperature. This study focused on how different bacterial strains from distinct isolation sources of both *V. parahaemolyticus* and *V. vulnificus* interact with common marine plastics, such as low-density polyethylene, polypropylene, and polystyrene, under different temperatures.

Bacterial cells have been shown to attach quicker and to grow and develop biofilms more rapidly on hydrophobic surfaces like plastics compared with hydrophilic surfaces like glass (Donlan, 2002). Our study further suggests plastic to be a more favorable substrate on average than glass for both *Vibrio* species at all temperatures tested under 24 h (Figure 1). Our study also indicates and further strengthens the assumption that *Vibrio* are early colonizers of plastics, especially LDPE, PP, and PS, as both *V. parahaemolyticus* and *V. vulnificus* were able to colonize and develop biofilms on these plastics within 24 h (Harrison et al., 2014; Kesy et al., 2021). Interestingly, most individual isolates besides *V. parahaemolyticus* ATCC17802 produced greater biofilm formation at lower temperature (25°C) compared to higher temperatures (30 and 35°C). This is in accordance with studies that have reported both *V. parahaemolyticus* and *V. vulnificus* biofilm growth in 96-well microplates under different temperature conditions (Han et al., 2016; Çam et al., 2019; Billaud et al., 2022). This suggests that *Vibrio* may produce greater amounts of biofilm as a survival mechanism in response to lower temperatures in the marine environment. However, when environmental conditions become more suitable and warmer, cells might be dispersing from these biofilms and contributing to higher planktonic cell concentrations (Townsley and Yildiz, 2015; Guilhen et al., 2017; Sheikh et al., 2022). In the context of climate change and public health, warming waters could be contributing to potentially higher exposure risk by this increased *Vibrio* biofilm dispersal leading to higher planktonic cell concentrations (Deeb et al., 2018).

The genus *Vibrio* has been reported to have “feast or famine” growth strategies, and the introduction of a new surface into a marine environment may provide a colonization opportunity niche which *Vibrio* rapidly respond to (Gilbert et al., 2012; Takemura et al., 2014; Westrich et al., 2016). However, while it did appear from our study that specific surface type could influence the colonization and biofilm development over a 24-h period, our study only observed *V. parahaemolyticus* and *V. vulnificus* colonization and biofilm development over 24-h on each individual plastic, so this process might be more undirected and driven by the colonization opportunity of a new surface. As there were visually observed differences in substrate flotation behavior and the substrates tested were confirmed to be different in specific density (Table 2), floatation behavior could also influence the adhesion of *Vibrio* species and, consequently, the production of biofilm on these substrates, especially in the context of *in situ* marine environments. This is important to note, as many studies have focused mainly on lower specific density plastics on the surface of marine environments as these plastic types are more easily observed, and not on plastics with higher specific density properties or on plastics that have lost buoyancy due to biofouling that are found at greater depths and in sediment (Zettler et al., 2013; Cózar et al., 2014; Van Sebille et al., 2015; Kirstein et al., 2016; Laverty et al., 2020; Delacuvellerie et al., 2022). While these two *Vibrio* species have been found in the ‘Plastisphere’ on the commonly occurring marine plastics assessed in the present study, studies on other *Vibrio* species colonization and biofilm development on different plastics, synthetic and organic polymers, and other substrate surfaces are still lacking both *in vitro* and *in vivo*.

There is high strain variability within *Vibrio* species in growth and biofilm formation. Strain variability has not been closely examined in plastic colonization (Whiting and Golden, 2002; Han et al., 2016; Odeyemi and Ahmad, 2017; Song et al., 2017; Çam and Brinkmeyer, 2020). *V. parahaemolyticus* human isolated strain ATCC17802 had the significantly greatest (*p* ≤ 0.01) biofilm formation on LDPE and PP compared to GL and compared to the other *V. parahaemolyticus* strains tested, especially at 30 and 35°C. Song et al. (2017) have also reported that pathogenic strains of *V. parahaemolyticus* form greater biofilms than non-pathogenic strains. This strain is positive for the *trh* gene, a known virulence factor, signifying that known *V. parahaemolyticus* human pathogenic strains can adequately colonize, and have considerable biofilm formation on plastics in a 24-h period, especially in warmer temperatures. Interestingly, without adjusting the CFUs per mm^2^ of surface type, it was also found that all *V. parahaemolyticus* isolates’ CFU concentrations on all plastic types had above the threshold dose needed to be infectious in humans (≥ 10^5^ CFUs) at all temperatures tested (Marx et al., 2013).

*Vibrio vulnificus* seawater isolated strain vv155 had the highest biofilm formation on all plastics at 25°C and was significantly greater on PS compared to GL and compared to the other *V. vulnificus* strains tested. While seawater isolates are expected to be strong biofilm producers to survive harsh marine environmental conditions, the result that *V. vulnificus* human isolate ATCC27562 was not the highest biofilm former was surprising. It was expected that human isolates would have the highest biofilm production between all isolate sources due to being isolated from the more stressful environment of the human host. However, research conducted by Çam and Brinkmeyer (2020) revealed that both clinical and environmental strains of *V. vulnificus* formed greater biofilms at lower temperatures. While the *V. vulnificus* human isolated strain ATCC27562 did not have the highest biofilm formation on plastics compared to this *V. vulnificus* water isolate, it cannot be ruled out that potential human pathogenic strains have higher colonization ability of plastic materials. This is especially apparent as the vv155 strain has a 16S rRNA designated type B genotype, which has a strong association with clinical strains, meaning that it may have a high level of virulence in humans (Nilsson et al., 2003). While type A strains are more environmentally associated, infections in humans from type A have still been reported, and been shown to be more virulent in mice (LD_50_ = 10^5^–10^6^ CFU) when compared to type B strains (LD_50_ = 10^8^ CFU; Amaro, 1992; Amaro and Biosca, 1996; Nilsson et al., 2003; Drake et al., 2007; Jones and Oliver, 2009; Çam et al., 2019; Wu et al., 2022). Only the *V. vulnificus* ATCC33147 type B strain CFUs on LDPE and PS had above the considered threshold LD_50_ dose of 10^5^–10^6^ CFUs (without adjusting the CFUs per mm^2^ of surface type) needed to be lethal in animals at all temperatures tested (Amaro and Biosca, 1996; Jeong and Satchell, 2012; Marx et al., 2013).

Biofilm biomass on substrate surfaces consists of the bacteria cells and their self-secreted extracellular polymeric substances (EPS) which are mainly comprised of biopolymers such as polysaccharides, proteins, and extracellular DNA (eDNA; Flemming, 2016; Decho and Gutierrez, 2017). These three major components of the EPS matrix contribute specific roles in biofilm formation, such as attachment and structural integrity (Dragoš and Kovács, 2017). The biofilm component dry mass, biochemical characteristics, and concentrations of EPS vary depending on the bacterial species and the environment in which the biofilm was grown/formed (Vu et al., 2009; Wagner et al., 2009; Villeneuve et al., 2011; Kavita et al., 2013). It is important to note that the dry cell and crude EPS mass and EPS biochemical concentrations obtained in this study might be underestimations of the total amount on the substrates tested, as portions of the weights and concentrations obtained from substrates might be lost during processing, and largely depend on the biofilm removal method, its removal efficiency, and EPS biochemical characterization treatments. Regardless, our study still observed a moderately positive correlation between the mean pooled dry biofilm biomass weight recovered from slide coupons and biofilm biomass from crystal violet staining of disc coupons, strengthening the assumption that crystal violet staining is an accurate method in estimating total biofilm biomass on substrates. Understanding the role of biochemical components in EPS may provide a further understanding of biofilm formation mechanisms of *V. parahaemolyticus* and *V. vulnificus* in their attachment to plastic substrates.

The quantitative analysis of the EPS from *V. parahaemolyticus* and *V. vulnificus* showed that extracellular proteins were the main component of EPS by mass of the mature biofilms on all plastic types, followed by carbohydrates then eDNA (Figure 4). These results suggested that extracellular proteins and carbohydrates were the main key components of the biofilm matrix of both species on plastics. These results are consistent with Li et al. (2020), which found extracellular proteins and carbohydrates were the main components of mature *V. parahaemolyticus* biofilms. To the best of our knowledge, this is one of the first studies to quantify and characterize *V. vulnificus* EPS and its overall biochemical characteristics, especially on plastics, compared to previous studies that focused more on genes that were correlated with biofilm formation (Joseph and Wright, 2004; Grau et al., 2008; Kim et al., 2011; Lee et al., 2013). However, as these three biochemical components did not quite equal 100% of the dry EPS mass of both species across all substrate types, there might be other smaller components that may be part of the EPS like metals, and further analysis is needed to confirm this in addition to identifying specific proteins and carbohydrates that make up both species EPS on plastics (Jiao et al., 2010).

The hydrophobicity of bacteria may differ between the strains of a species and may change in response to changes in environmental conditions (temperature, nutrient availability, etc.), growth phases, and growth state (planktonic vs biofilm; Nwanyanwu et al., 2012). The present results also indicate this, as both *V. parahaemolyticus* and *V. vulnificus* strains possess wide differences in their hydrophobicity in the planktonic state at different temperatures. Both *Vibrio* species were considered hydrophobic, with *V. vulnificus* being more hydrophobic than *V. parahaemolyticus* at all temperatures tested, especially at 30 and 35°C (19 and 16% more hydrophobic, respectively) based on their adhesion to p-xylene, a hydrocarbon (Supplementary Table S6). All individual strains were considered hydrophobic at all temperatures tested (Figure 5). Only one *V. parahaemolyticus* strain (ATCC43996) had strong adhesion to p-xylene and thus was considered highly hydrophobic at 25 and 30°C, while all *V. vulnificus* strains had strong adhesion to p-xylene at 25°C, and *V. vulnificus* strain ATCC33147 exhibited strong adhesion to hydrocarbons at all temperatures tested (Figure 5). These results confirm the high variability of the hydrophobicity of *Vibrio* species and strains’ planktonic cells, and that different temperatures can influence the degree of hydrophobicity (Lee and Yii, 1996; Wong and Chang, 2005; Mizan et al., 2016). The development of specific adaptive mechanisms of *Vibrio* to the toxicity and low bioavailability of these plastic substrates could contribute to the modification of its cell surface hydrophobicity to permit direct hydrophobic-hydrophobic interactions with these plastic substrates in initial colonization. This could lead to potential biodegradation of plastics as it has been reported that adequate hydrophobic/hydrophilic properties of bacteria can contribute to degradation of hydrocarbons (Krasowska and Sigler, 2014).

Taken together, these results indicate that different strain types of *V. parahaemolyticus V. vulnificus* can rapidly and adequately form biofilms with high viable cell concentrations on different plastic material types *in vitro*. However, this colonization process is highly variable and depends on species, strain, and plastic type, especially under different temperatures. Further studies are needed to compare these *Vibrio in vitro* plastic colonization processes to those found in the natural marine environment. While seawater surface temperature is monitored as it is predictive for *Vibrio* growth, this monitoring only accounts for planktonic cell growth and biofilms must also be included in monitoring. Seafood is already screened and tested for potential *Vibrio* contamination, but additional screening for plastic particles in seafood must also be considered as humans are likely to be frequently exposed to plastics particles as they have been found in high concentrations in commercially harvested seafood (Wu et al., 2019; Curren et al., 2020; Nicole, 2021). The present results highlight the ability of *Vibrio* species to form biofilms on plastics, and may need to be incorporated into forecast models for *Vibrio* risk to better predict potential human exposure to pathogenic *Vibrios*, especially under climate change scenarios (Jacobs et al., 2014; Deeb et al., 2018; Ferchichi et al., 2021). Lastly, as both *V. parahaemolyticus* and *V. vulnificus* have been demonstrated to rapidly colonize plastics, their ability to utilize and degrade LDPE, PP, and PS also needs to be further explored (Obuekwe et al., 2009; Heipieper et al., 2010; Harrison et al., 2014; Raghul et al., 2014).

## Data availability statement

The original contributions presented in the study are included in the article/Supplementary material, further inquiries can be directed to the corresponding author.

## Author contributions

RL, AD, and RN conceived and designed the study, analyzed the data, corrected the draft, built the final version of the manuscript, and read and approved the submitted version. RL, KCV, AC, KA, and GA performed the lab experiments. LX and GC performed statistical analyses. RL wrote the first draft of the manuscript. All authors contributed to the article and approved the submitted version.

## Funding

This work has been funded by the NIEHS Center for Oceans and Human Health and Climate Change Interactions at the University of South Carolina (grant #P01ES028942).

## Conflict of interest

The authors declare that the research was conducted in the absence of any commercial or financial relationships that could be construed as a potential conflict of interest.

## Publisher’s note

All claims expressed in this article are solely those of the authors and do not necessarily represent those of their affiliated organizations, or those of the publisher, the editors and the reviewers. Any product that may be evaluated in this article, or claim that may be made by its manufacturer, is not guaranteed or endorsed by the publisher.
