# Supplementary material for: Vibrio parahaemolyticus and Vibrio vulnificus in vitro colonization on plastics influenced by temperature and strain variability
Source: Front Microbiol. 2023 Jan 10;13:1099502. doi: 10.3389/fmicb.2022.1099502 (PMC9871911; doi:10.3389/fmicb.2022.1099502)
Supplement: Supplementary file 1 [file Data_Sheet_1.docx]

Supplementary Material

# Supplementary Data

Bacterial Isolation and Identification

*V. vulnificus* strain vv155 was isolated from seawater samples from a past study and was provided as a gift from NOAA to be used as the *V. vulnificus* environmental strain in this study. Seawater samples were collected in 1L sterile, polypropylene containers and diluted and filtered through 0.22 0.22µm nitrocellulose membrane filter. Filters were then placed on two types of selective media, CPC+ and CHROMagar™ *Vibrio*. Presumptive *V. vulnificus* strains were then identified by species using a TaqMan based real-time PCR targeting the hemolysin *A* gene (vvhA). The characterization of each isolate as type A (environmental), Type B (clinical) or Type AB was performed using qPCR according to a protocol developed by Vickery et al., 2007. Dr. Joanna Mort (James Madison University), Dr. Jerold Dickerson (James Madison University) and Dr. Janet Moore (NOAA) all contributed to this past isolation and characterization study.

*V. parahaemolyticus* strain vpC12 was isolated from seawater samples to be used as the *V. parahaemolyticus* environmental strain for this study. Water samples (800 mL) were collected using an ISCO 6700 autosampler (ISCO, Lincoln, NE, USA) for 24 hours during low and high tides at the Oyster Landing Dock at the Baruch Marine Research Laboratory, Georgetown, SC (33°20'58" N 79°11'20" W). Water samples from each tidal period were combined and homogenized. One liter of the combined sample was filtered through a 0.22µm polyethersulfone (PES) membrane filter (Thermo Sci., Waltham, MA, USA) to concentrate the microbial community in the 0.22µm filter. The microbial community collected in the filter was reconstituted in 20 mL of the unfiltered combined samples. The reconstituted sample was diluted using serial dilutions. From each dilution, 550µL of the sample was grown in selective and differential media, thiosulfate citrate bile sucrose (TCBS, Sigma-Aldrich, St. Louis, MO, USA), and CHROMagar™ *Vibrio* (Kanto Chemical, Chuo-Ku, Tokyo, Japan). The green colonies in TCBS and the purple/mauve colonies in CHROMagar™ *Vibrio* were identified as presumptive *Vibrio*. All presumptive *Vibrio* strains then were identified by species using multiplex polymerase chain reaction (PCR) using primers developed by Kim et al., 2015 to identify the *tlh* species marker gene. [Accession number: OP912877]

Table S1. Effect of temperature on mean biofilm production^1^ per mm^2^ on different plastic surface types^2^ by *V. parahaemolyticus* and *V. vulnificus* individual strains compared to glass and 25°C.

| **Species** | **Strain** | **Surface Type** | **25°C (± SD)** | **30°C (± SD)** | **35°C (± SD)** | **Surface *p*-value** |
| --- | --- | --- | --- | --- | --- | --- |
| *V. parahaemolyticus* | ATCC17802 | GL | 3.35E-04 (1.58E-04) | 6.84E-04 (2.35E-04) | 1.32E-03 (6.67E-04) |  |
|  |  | LDPE | 2.56E-03* (1.95E-03) | 5.98E-03*$ (1.36E-03) | 5.75E-03*$ (7.23E-04) | <0.01 |
|  |  | PP | 1.87E-03* (1.84E-03) | 6.04E-03*$ (8.07E-04) | 5.83E-03*$ (4.71E-04) | <0.01 |
|  |  | PS | 7.27E-04* (6.91E-04) | 2.02E-03*$ (3.14E-04) | 1.15E-03*$ (3.86E-04) | <0.05 |
| **Temperature *p*-value** |  |  |  | <0.01 | <0.01 |  |
| *V. parahaemolyticus* | ATCC43996 | GL | 4.08E-04 (2.89E-04) | 2.61E-04 (1.60E-04) | 2.05E-04 (1.27E-04) |  |
|  |  | LDPE | 1.31E-03* (1.53E-04) | 7.24E-04*x (3.79E-04) | 6.87E-04*x (4.77E-04) | <0.01 |
|  |  | PP | 8.91E-04* (8.00E-04) | 3.40E-04*x (1.44E-04) | 3.75E-04*x (1.42E-04) | <0.01 |
|  |  | PS | 1.01E-03* (9.21E-04) | 8.46E-04*x (2.07E-04) | 6.84E-04*x (2.84E-04) | <0.01 |
| **Temperature *p*-value** |  |  |  | <0.01 | <0.01 |  |
| *V. parahaemolyticus* | vpC12 | GL | 3.09E-04 (2.08E-04) | 3.06E-04 (1.46E-04) | 6.99E-04 (3.06E-04) |  |
|  |  | LDPE | 8.06E-04 (3.92E-04) | 1.58E-04x (5.97E-05) | 6.40E-04 (1.37E-04) | 0.86 |
|  |  | PP | 5.65E-04 (3.66E-04) | 7.88E-05x (6.34E-05) | 5.37E-04 (8.90E-05) | 0.42 |
|  |  | PS | 9.02E-04 (2.63E-04) | 4.34E-04x (2.63E-04) | 7.00E-04 (2.95E-05) | 0.28 |
| **Temperature *p*-value** |  |  |  | <0.05 | 0.84 |  |
| *V. vulnificus* | ATCC27562 | GL | 2.31E-03 (5.94E-04) | 1.37E-03 (8.01E-04) | 6.41E-04 (2.23E-04) |  |
|  |  | LDPE | 2.08E-03 (2.05E-03) | 8.49E-04x (6.25E-04) | 1.38E-03x (6.46E-04) | 0.99 |
|  |  | PP | 1.93E-03 (1.52E-03) | 4.23E-04x (2.13E-04) | 5.24E-04x (3.12E-04) | 0.46 |
|  |  | PS | 3.67E-03 (1.09E-03) | 2.18E-03x (6.19E-04) | 1.53E-03x (1.94E-04) | 0.31 |
| **Temperature *p*-value** |  |  |  | <0.05 | <0.05 |  |
| *V. vulnificus* | ATCC33147 | GL | 1.96E-03 (5.28E-04) | 4.02E-04 (1.30E-04) | 2.59E-04 (8.0E-05) |  |
|  |  | LDPE | 2.01E-03 (1.63E-03) | 1.20E-03x (5.12E-04) | 1.24E-03x (3.13E-04) | 0.09 |
|  |  | PP | 1.18E-03 (8.50E-04) | 7.34E-04x (4.99E-04) | 3.49E-04x (2.81E-04) | 0.99 |
|  |  | PS | 4.00E-03* (1.31E-03) | 1.59E-03*x (3.53E-04) | 1.08E-03*x (4.40E-04) | <0.05 |
| **Temperature *p*-value** |  |  |  | <0.05 | <0.01 |  |
| *V. vulnificus* | vv155 | GL | 2.90E-03 (3.80E-04) | 1.18E-03 (4.33E-04) | 1.95E-03 (3.33E-04) |  |
|  |  | LDPE | 4.66E-03 (1.68E-03) | 3.25E-03 (5.19E-04) | 4.31E-03 (7.55E-04) | 0.06 |
|  |  | PP | 2.27E-03 (1.17E-03) | 2.75E-03 (1.60E-03) | 2.35E-03 (1.75E-04) | 0.99 |
|  |  | PS | 5.09E-03* (1.62E-03) | 4.54E-03* (1.14E-03) | 3.15E-03* (2.43E-04) | <0.05 |
| **Temperature *p*-value** |  |  |  | 0.34 | 0.54 |  |

^1^Optical density at 570 nm

^2^Biomass values divided by total coupon surface area (405 mm^2^)

(± SD) Standard deviation

(*) Significant increase of biofilm production on plastic surface type compared to glass

($) Significant increase of biofilm production on surface at this temperature compared to 25°C

(x) Significant decrease of biofilm production on surface at this temperature compared to 25°C

Table S2. Statistical comparison of *V. parahaemolyticus* and *V. vulnificus* animal and seawater isolates’ biofilm production compared to human isolated strains

| **Species** | **Isolation Source** | **Strain** | **Coefficient** | ***p*** |
| --- | --- | --- | --- | --- |
| *V. parahaemolyticus* | Animal | ATCC43996 | -0.89* | <0.001 |
|  | Seawater | vpC12 | -0.94* | <0.001 |
| *V. vulnificus* | Animal | ATCC33147 | -0.09 | 0.99 |
|  | Seawater | vv155 | 0.65* | <0.001 |

*Significant increase (+) /decrease (-) in biofilm production

Table S3. Statistical comparison of *V. parahaemolyticus* biofilm production on different surface types compared to glass

| **Species** | **Surface Type** | **Coefficient** | ***p*** |
| --- | --- | --- | --- |
| *V. parahaemolyticus* | LDPE | 0.63 | 0.29 |
|  | PP | 0.53 | 0.72 |
|  | PS | 0.17 | 0.99 |

*Significant increase (+) /decrease (-) in biofilm production

Table S4. Statistical comparison of *V. vulnificus* biofilm production on different surface types compared to glass

| **Species** | **Surface Type** | **Coefficient** | ***p*** |
| --- | --- | --- | --- |
| *V. vulnificus* | LDPE | 0.35 | 0.14 |
|  | PP | -0.02 | 0.99 |
|  | PS | 0.62* | <0.001 |

*Significant increase (+) /decrease (-) in biofilm production

Table S5. Statistical comparison of *V. parahaemolyticus* and *V. vulnificus* biofilm production at higher temperatures (30, 35°C) compared to lower temperature (25°C)

| **Species** | **Temperature** | **Coefficient** | ***p*** |
| --- | --- | --- | --- |
| *V. parahaemolyticus* | 30°C | 0.20 | 0.99 |
|  | 35°C | 0.23 | 0.99 |
| *V. vulnificus* | 30°C | -0.48* | <0.01 |
|  | 35°C | -0.51* | <0.01 |

*Significant increase (+) /decrease (-) in biofilm production

Table S6. Effect of temperature on *V. parahaemolyticus* and *V. vulnificus* individual strain CFUs^1^ per mm^2^ on different plastic surface types^2^ by compared to glass and 25°C.

| **Species** | **Strain** | **Surface Type** | **25°C (± SD)** | **30°C (± SD)** | **35°C (± SD)** | **Surface *p*-value** |
| --- | --- | --- | --- | --- | --- | --- |
| *V. parahaemolyticus* | ATCC17802 | GL | 2.31 (1.42) | 4.23 (1.01) | 3.16 (2.41) |  |
|  |  | LDPE | 3.81* (2.24) | 4.23*$ (3.05) | 4.31*$ (3.21) | <0.001 |
|  |  | PP | 3.36* (1.79) | 4.18*$ (3.09) | 4.31*$ (3.59) | <0.001 |
|  |  | PS | 3.56* (2.15) | 3.05*$ (3.35) | 3.88*$ (2.87) | <0.01 |
| **Temperature *p*-value** |  |  |  | <0.05 | <0.01 |  |
| *V. parahaemolyticus* | ATCC43996 | GL | 2.97 (1.85) | 3.28 (2.40) | 2.60 (1.56) |  |
|  |  | LDPE | 3.76 (2.41) | 3.39 (2.44) | 3.36 (2.06) | 0.19 |
|  |  | PP | 3.43 (2.72) | 2.57 (2.39) | 3.26 (2.39) | 0.35 |
|  |  | PS | 3.73 (3.06) | 2.55 (1.48) | 2.75 (1.66) | 0.99 |
| **Temperature *p*-value** |  |  |  | 0.20 | 0.12 |  |
| *V. parahaemolyticus* | vpC12 | GL | 2.54 (1.43) | 3.06 (1.50) | 3.31 (2.37) |  |
|  |  | LDPE | 3.98 (3.40) | 2.39 (2.53) | 2.97 (2.23) | 0.76 |
|  |  | PP | 3.65 (2.58) | 3.06 (1.51) | 2.53 (1.12) | 0.99 |
|  |  | PS | 3.73 (2.54) | 4.23 (1.90) | 2.97 (2.23) | 0.99 |
| **Temperature *p*-value** |  |  |  | 0.13 | 0.29 |  |
| *V. vulnificus* | ATCC27562 | GL | 4.16 (3.22) | 3.98 (3.31) | 2.78 (1.78) |  |
|  |  | LDPE | 3.44 (2.77) | 3.59 (2.88) | 4.02 (3.42) | 0.99 |
|  |  | PP | 3.73 (2.82) | 3.12 (1.94) | 3.95 (3.21) | 0.99 |
|  |  | PS | 4.19 (3.58) | 4.08 (2.63) | 3.67 (3.05) | 0.99 |
| **Temperature *p*-value** |  |  |  | 0.99 | 0.96 |  |
| *V. vulnificus* | ATCC33147 | GL | 3.53 (2.53) | 2.27 (1.54) | 2.45 (1.64) |  |
|  |  | LDPE | 4.71* (3.37) | 4.49* (3.93) | 3.75* (2.37) | <0.05 |
|  |  | PP | 2.31 (1.03) | 2.28 (1.71) | 2.78 (1.80) | 0.99 |
|  |  | PS | 4.56 (3.65) | 3.57 (2.56) | 3.72 (2.78) | 0.06 |
| **Temperature *p*-value** |  |  |  | 0.18 | 0.18 |  |
| *V. vulnificus* | vv155 | GL | 3.50 (3.24) | 4.34 (3.49) | 3.87 (3.55) |  |
|  |  | LDPE | 3.42 (3.16) | 4.40 (3.32) | 4.75 (3.79) | 0.99 |
|  |  | PP | 4.07 (3.15) | 3.71 (2.30) | 4.61 (3.67) | 0.99 |
|  |  | PS | 3.52 (3.63) | 4.76 (3.38) | 4.57 (4.25) | 0.99 |
| **Temperature *p*-value** |  |  |  | 0.13 | 0.07 |  |

^1^CFU values were log transformed

^2^CFU values divided by total coupon surface area (405 mm^2^)

(± SD) Standard deviation

(*) Significant increase of CFUs on plastic surface type compared to glass

($) Significant increase of CFUs on this surface at this temperature compared to 25°C

Table S7. Statistical comparison of *V. parahaemolyticus* and *V. vulnificus* animal and water isolates’ CFUs compared to human isolated strains

| **Species** | **Isolation Source** | **Strain** | **Coefficient** | ***p*** |
| --- | --- | --- | --- | --- |
| *V. parahaemolyticus* | Animal | ATCC43996 | -2.90E+06* | <0.01 |
|  | Seawater | vpC12 | -2.85E+06* | <0.01 |
| *V. vulnificus* | Animal | ATCC33147 | 1.63E+06 | 0.99 |
|  | Seawater | vv155 | 6.18E+06* | <0.05 |

*Significant increase (+) /decrease (-) in CFU concentration

Table S8. Statistical comparison of *V. parahaemolyticus* CFUs on different surface types compared to glass

| **Species** | **Surface Type** | **Coefficient** | ***p*** |
| --- | --- | --- | --- |
| *V. parahaemolyticus* | LDPE | 2.61E+06 | 0.14 |
|  | PP | 1.98E+06 | 0.13 |
|  | PS | 1.48E+06 | 0.42 |

Table S9. Statistical comparison of *V. vulnificus* CFUs on different surface types compared to glass

| **Species** | **Surface Type** | **Coefficient** | ***p*** |
| --- | --- | --- | --- |
| *V. vulnificus* | LDPE | 5.78E+06 | 0.99 |
|  | PP | 5.96E+05 | 0.99 |
|  | PS | 5.16E+06 | 0.99 |

Table S10. Statistical comparison of *V. parahaemolyticus* and *V. vulnificus* CFUs at higher temperatures (30, 35°C) compared to lower temperature (25°C)

| **Species** | **Temperature** | **Coefficient** | ***p*** |
| --- | --- | --- | --- |
| *V. parahaemolyticus* | 30°C | 3.70E+05 | 0.99 |
|  | 35°C | 3.97E+05 | 0.99 |
| *V. vulnificus* | 30°C | 7.25E+05 | 0.99 |
|  | 35°C | 9.55E+05 | 0.99 |

Table S11. *V. parahaemolyticus* and *V. vulnificus* extracellular polymeric substance biochemical concentrations (µg/mL) and corresponding starting EPS weight (µg) from 10 pooled samples.

| **Species** | **Strain** | **Surface Type** | **Protein** | **EPS** | **Carbohydrates** | **EPS** | **eDNA** | **EPS** |
| --- | --- | --- | --- | --- | --- | --- | --- | --- |
| *V. parahaemolyticus* | ATCC17802 | LDPE | 150 | 200 | 145 | 900 | 0.94 | 100 |
|  |  | PP | 153 | 200 | 146 | 700 | 1.25 | 100 |
|  |  | PS | 151 | 200 | 18 | 100 | 0.36 | 100 |
| *V. vulnificus* | vv155 | LDPE | 80 | 100 | 17 | 100 | 1.26 | 100 |
|  |  | PP | 83 | 100 | 26 | 300 | 0.89 | 100 |
|  |  | PS | 140 | 200 | 52 | 200 | 0.94 | 100 |

Table S12. *V. parahaemolyticus* and *V. vulnificus* individual strain mean adherence to p-xylene (%) at different temperatures.

| **Species** | **Strain** | **25°C (± SD)** | **30°C (± SD)** | **35°C (± SD)** |
| --- | --- | --- | --- | --- |
| *V. parahaemolyticus* | ATCC17802 | 55% (5) | 49% (4) | 49% (6) |
|  | ATCC43996 | 83% (2) | 72% (2) | 64% (4) |
|  | vpC12 | 58% (4) | 36% (8) | 36% (6) |
| **Correlation between total plastic surface biofilm biomass (*r*)** |  | -0.19 | -0.06 | -0.04 |
| *V. vulnificus* | ATCC27562 | 73% (5) | 69% (3) | 62% (2) |
|  | ATCC33147 | 76% (10) | 77% (1) | 70% (8) |
|  | vv155 | 71% (1) | 66% (4) | 66% (2) |
| **Correlation between total plastic surface biofilm biomass (*r*)** |  | -0.47 | -0.60 | -0.08 |

(± SD) Standard deviation

Table S13. *V. parahaemolyticus* and *V. vulnificus* species mean adherence to p-xylene (%) at different temperatures.

| **Species** | **25°C** | **30°C** | **35°C** |
| --- | --- | --- | --- |
| *V. parahaemolyticus* | 65% | 52% | 50% |
| *V. vulnificus* | 73% | 71% | 66% |

# Supplementary Figures and Tables


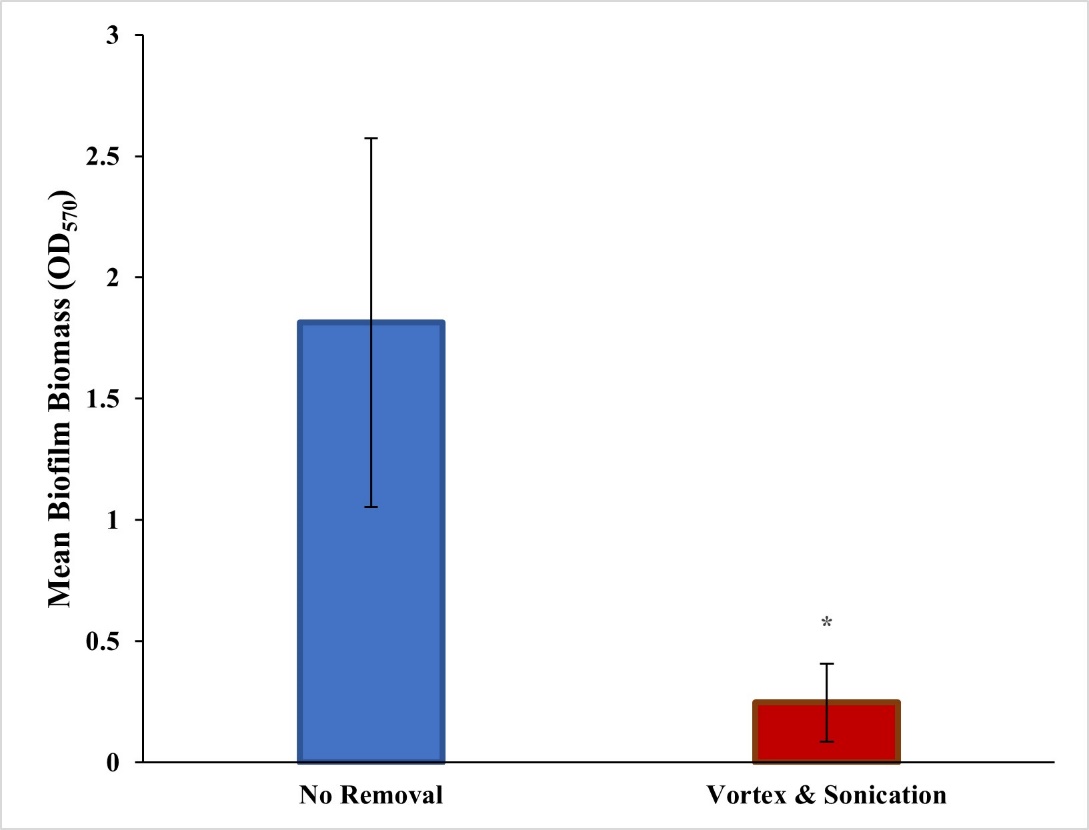


Figure S1. Assessment of mean *V. vulnificus* (vv155) biofilm biomass removal by vortex & sonication method of combined surface types. * = significant removal of biofilm (α = 0.05) as calculated by t-test.


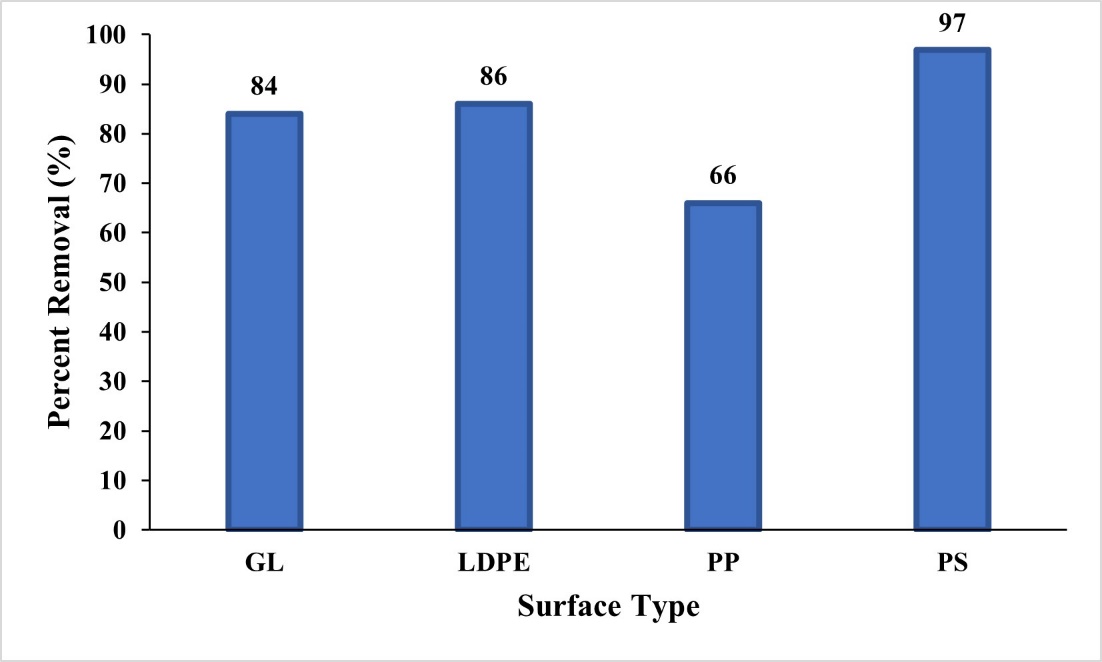


Figure S2. Assessment of mean *V. vulnificus* (vv155) biofilm biomass removal by vortex and sonication method of individual surface types.

Table S14. Assessment of vortex and sonication biofilm biomass^1^ removal method

|  | **GL (± SD)** | **LDPE (± SD)** | **PP (± SD)** | **PS (± SD)** | **Mean Combined Biofilm Biomass (± SD)** | ***p*** |
| --- | --- | --- | --- | --- | --- | --- |
| **Control** | 1.52 (0.25) | 2.74 (0.07) | 0.95 (0.2) | 2.02 (0.13) | 1.81 (0.76) |  |
| **Vortex & Sonication** | 0.24 (0.5) | 0.39 (0.04) | 0.32 (0.07) | 0.02 (0.07) | 0.24 (0.16) | <0.05 |
| **Percent Removal** | 84% | 86% | 66% | 97% | 86% |  |

^1^Optical density at 570 nm

(± SD) Standard deviation


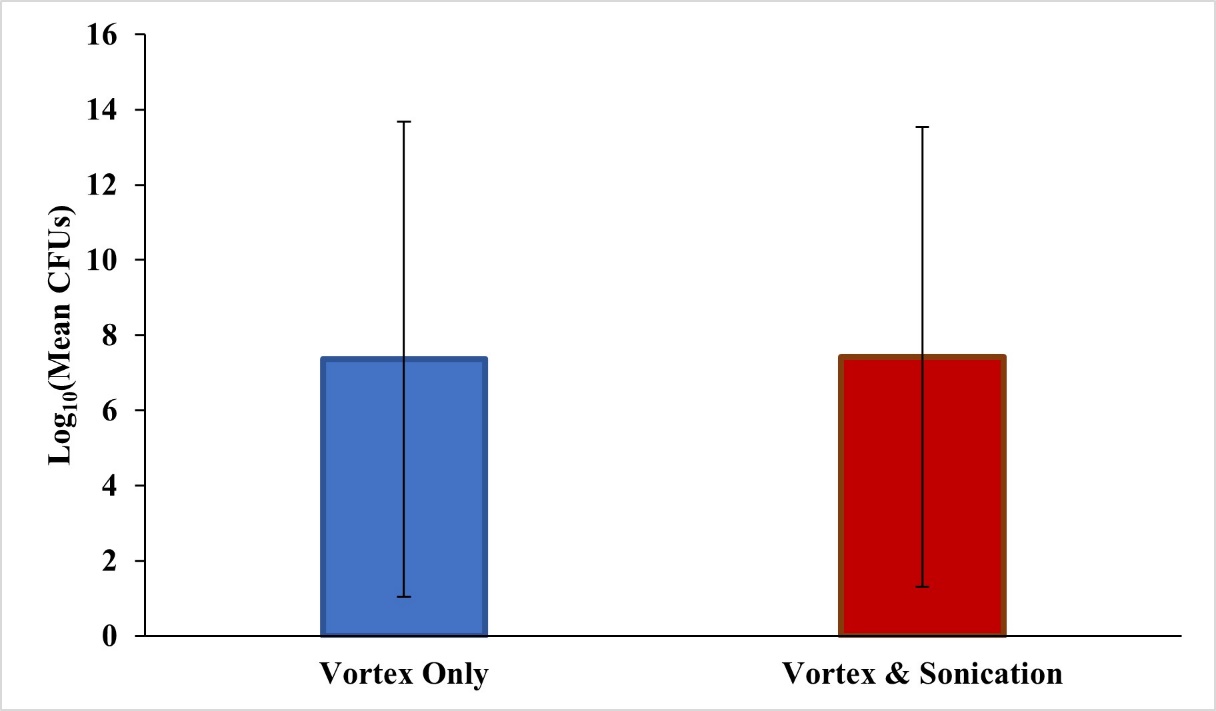


Figure S3. Assessment of sonication on *V. vulnificus* (vv155) cell viability

Table S15. Confirmation that sonication has no significant effect on cell viability

|  | **Vortex Only**  **(± SD)** | **Vortex & Sonication**  **(± SD)** | ***p*** |
| --- | --- | --- | --- |
| **Mean CFUs** | 2.30E+07 (2.09E+06) | 2.62E+07 (1.29E+06) | 0.07 |


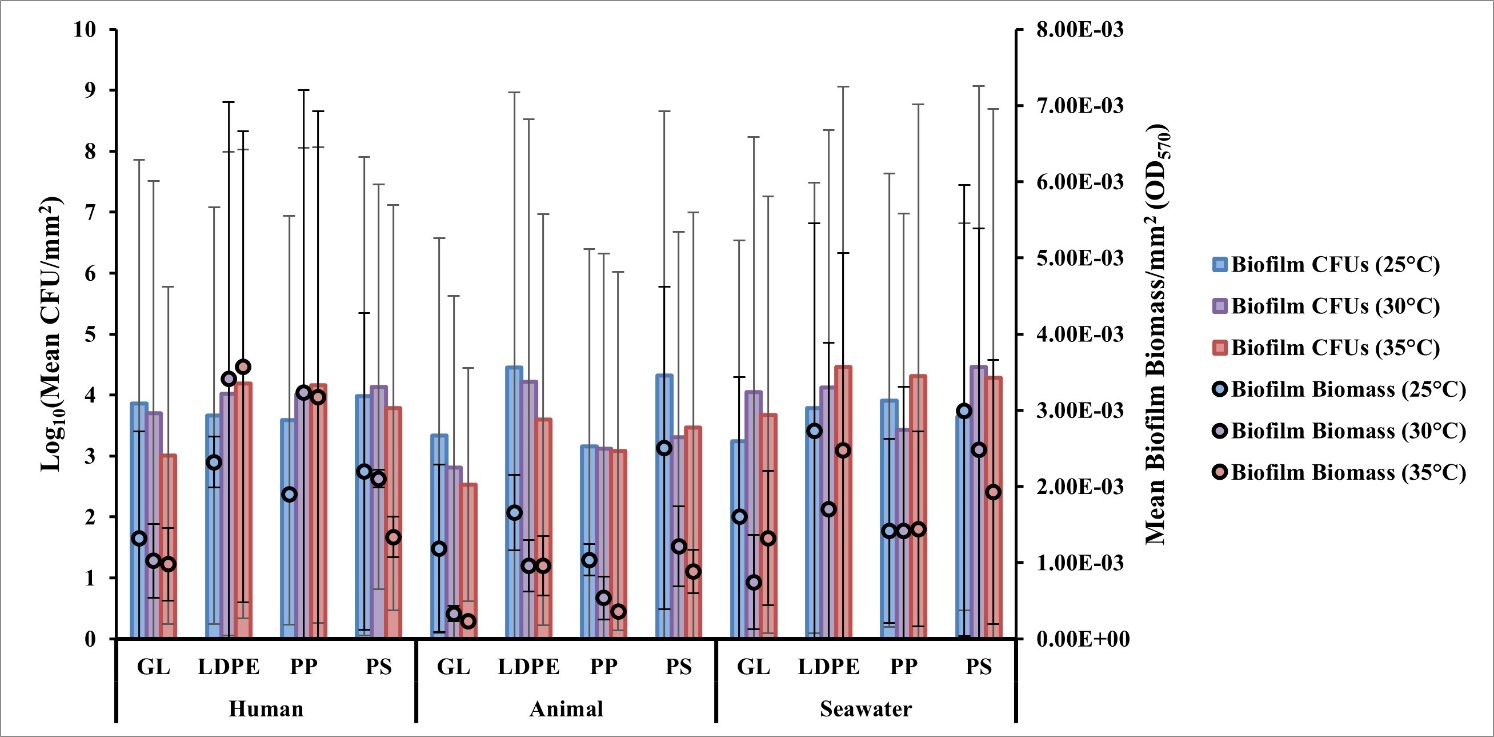


Figure S4. Comparison of the effect of temperature on biofilm production on different plastic surface types by different *Vibrio* isolation sources.
